# Novel ^177^Lu-Labeled [Thz^14^]Bombesin(6–14) Derivatives with Low Pancreas Accumulation for Targeting Gastrin-Releasing Peptide Receptor-Expressing Cancer

**DOI:** 10.3390/ph18040449

**Published:** 2025-03-23

**Authors:** Lei Wang, Devon E. Chapple, Hsiou-Ting Kuo, Sara Kurkowska, Ryan P. Wilson, Wing Sum Lau, Pauline Ng, Carlos Uribe, François Bénard, Kuo-Shyan Lin

**Affiliations:** 1 Department of Molecular Oncology, BC Cancer Research Institute, Vancouver, BC V5Z 1L3, Canada; lewang@bccrc.ca (L.W.); devchapple@gmail.com (D.E.C.); htkuo0325@gmail.com (H.-T.K.); rpwilson98@gmail.com (R.P.W.); wlau@bccrc.ca (W.S.L.); png@bccrc.ca (P.N.); fbenard@bccrc.ca (F.B.); 2 Department of Integrative Oncology, BC Cancer Research Institute, Vancouver, BC V5Z 1L3, Canada; skurkowska@bccrc.ca (S.K.); curibe@bccrc.ca (C.U.); 3 Department of Nuclear Medicine, Pomeranian Medical University, 70-204 Szczecin, Poland; 4 Department of Molecular Imaging and Therapy, BC Cancer, Vancouver, BC V5Z 4E6, Canada; 5 Department of Radiology, University of British Columbia, Vancouver, BC V5Z 1M9, Canada

**Keywords:** gastrin-releasing peptide receptor, single-photon emission computed tomography, lutetium-177, dosimetry, radioligand therapy

## Abstract

**Background/Objectives:** Gastrin-releasing peptide receptor is a promising target for cancer diagnosis and therapy. However, the high pancreas uptake of reported GRPR-targeted radioligands limits their clinical applications. Our group previously reported one ^68^Ga-labeled GRPR antagonist, [^68^Ga]Ga-TacsBOMB5 (^68^Ga-DOTA-Pip-[D-Phe^6^,NMe-Gly^11^,Leu^13^ψThz^14^]Bombesin(6–14)), and two agonists, [^68^Ga]Ga-LW01110 (^68^Ga-DOTA-Pip-[D-Phe^6^,Tle^10^,NMe-His^12^,Thz^14^]Bombesin(6–14)) and [^68^Ga]Ga-LW01142 (^68^Ga-DOTA-Pip-[D-Phe^6^,His^7^,Tle^10^,NMe-His^12^,Thz^14^]Bombesin(6–14)) showing minimal pancreas uptake. Thus, in this study, we prepared their ^177^Lu-labeled analogs, evaluated their therapeutic potentials, and compared them with the clinically evaluated [^177^Lu]Lu-AMBA. **Methods**: GRPR binding affinities were determined by in vitro competition binding assay using PC-3 prostate cancer cells. Longitudinal SPECT/CT imaging and ex vivo biodistribution studies were conducted in PC-3 tumor-bearing mice. Dosimetry data were calculated from the biodistribution results. **Results**: The K_i_(GRPR) values of Lu-TacsBOMB5, Lu-LW01110, Lu-LW01142, and Lu-AMBA were 12.6 ± 1.02, 3.07 ± 0.15, 2.37 ± 0.28, and 0.33 ± 0.16 nM, respectively. SPECT/CT images and biodistribution results demonstrated good tumor accumulation of [^177^Lu]Lu-TacsBOMB5, [^177^Lu]Lu-LW01110, and [^177^Lu]Lu-LW01142 at early time points with rapid clearance over time. The pancreas uptake of all three [Thz^14^]Bombesin(6–14)-derived ligands was significantly lower than that of [^177^Lu]Lu-AMBA at all time points. The calculated absorbed doses of [^177^Lu]Lu-TacsBOMB5, [^177^Lu]Lu-LW01110, and [^177^Lu]Lu-LW01142 in PC-3 tumor xenografts were 87.1, 312, and 312 mGy/MBq, respectively, higher than that of [^177^Lu]Lu-AMBA (79.1 mGy/MBq), but lower than that of the previously reported [^177^Lu]Lu-RM2 (429 mGy/MBq). **Conclusions**: Our data suggest that [^177^Lu]Lu-TacsBOMB5 and [^177^Lu]Lu-LW01142 reduce radiation exposure to the pancreas. However, further optimizations are needed for both radioligands to prolong their tumor retention and enhance treatment efficacy.

## 1. Introduction

Overexpressed in many solid malignancies, gastrin-releasing peptide receptor (GRPR) is a promising target for diagnosis and radioligand therapy of GRPR-expressing cancers [1,2,3,4,5]. GRPR was found overexpressed in 100% of early-stage prostate cancer tissues, around 60% of late-stage prostate cancer tissues, and 83.2% of ER-positive breast cancer tissues [5,6]. However, high pancreas uptake was observed for most of reported GRPR-targeted radiopharmaceuticals, including the clinically evaluated radiolabeled RM2, SB3, NeoB, and AMBA derivatives [7,8,9,10,11]. Although the absorbed dose limit of the pancreas for radiation therapy is still unclear [12,13], studies have shown that the pancreatic tissue may undergo structural and functional alterations with radiation doses reaching 30–40 Gy and the pancreas should be considered as an organ at risk (OAR) [14,15]. Thus, developing novel GRPR-targeted radioligands with low pancreas uptake is desirable to minimize potential toxicity.

Previously, our group developed one ^68^Ga-labeled GRPR antagonist ([^68^Ga]Ga-TacsBOMB5) [16] and two ^68^Ga-labeled GRPR agonists ([^68^Ga]Ga-LW01110 and [^68^Ga]Ga-LW01142) [17] for detecting GRPR-expressing cancer with positron emission tomography (PET). All three tracers showed high uptake in PC-3 tumor xenografts (11.4 to 15.7%ID/g) and much lower accumulation in the pancreas (1.98 to 8.99%ID/g) at 1 h post-injection, suggesting their potential for detecting GRPR-expressing cancer lesions [16,17]. Additionally, our findings indicate the potential of labeling these three candidates with an α- or β-emitter for radiotherapeutic applications owing to the lower radiation accumulation in healthy organs, particularly the pancreas.

**Figure 1:**
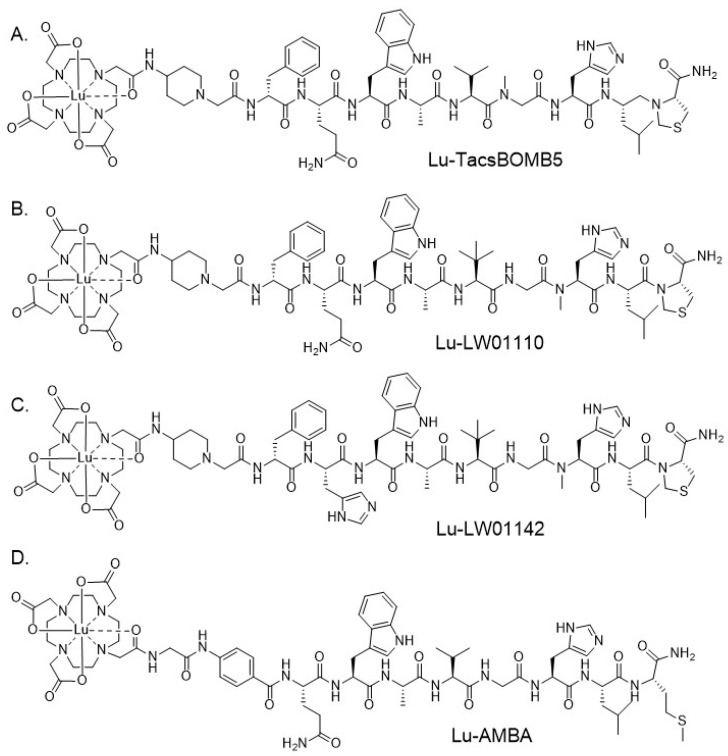
Chemical structures of (**A**) Lu-TacsBOMB5, (**B**) Lu-LW01110, (**C**) Lu-LW01142, and (**D**) Lu-AMBA.

Lutetium-177, a β-emitter (E_β(max)_ 497 keV (78.6%), E_β(max)_ 384 keV (9.1%), and E_β(max)_ 176 keV (12.2%); 6.65 days half-life), is widely used for radiotherapeutic applications and is stably chelated by the universal chelator, 1,4,7,10-tetraazacyclododecane-1,4,7,10-tetraacetic acid (DOTA) [18]. Several ^68^Ga-/^177^Lu-labeled theranostic agents have been used for cancer diagnosis and radiotherapy, such as [^68^Ga]Ga-/[^177^Lu]Lu-NeoB and [^68^Ga]Ga-/[^177^Lu]Lu-DOTA-TATE [19,20]. Therefore, for this study, we replaced ^68^Ga in our previously reported tracers with ^177^Lu to obtain [^177^Lu]Lu-TacsBOMB5 (Figure 1A), [^177^Lu]Lu-LW01110 (Figure 1B), and [^177^Lu]Lu-LW01142 (Figure 1C). We evaluated the potential of these ^177^Lu-labeled ligands as GRPR-targeted therapeutic agents by SPECT imaging and ex vivo biodistribution studies in GRPR-expressing tumor-bearing mice and dosimetry calculation. The results were compared with that obtained with the clinically evaluated GRPR agonist [^177^Lu]Lu-AMBA (Figure 1D).

## 2. Results

### 2.1. Peptide Synthesis and Radiolabeling

The Lu-complexed nonradioactive standards of TacsBOMB5, LW01110, LW01142, and AMBA were obtained in 45–88% yields with >97% purity (Table S1). The MS characterizations of Lu-TacsBOMB5, Lu-LW01110, Lu-LW01142, and Lu-AMBA are provided in Table S1 and Figures S1–S4. The ^177^Lu-labeled TacsBOMB5, LW01110, LW01142, and AMBA were obtained in 24–62% decay-corrected radiochemical yields with 165–348 GBq/µmol molar activity and >91% radiochemical purity. The good molar activity ranging from 165–348 GBq/μmol was suitable for preclinical studies in mice. HPLC conditions for their purification and quality control are provided in Table S2.

### 2.2. Binding Affinity, Agonist/Antagonist Characteristics, and Hydrophilicity

**Figure 2:**
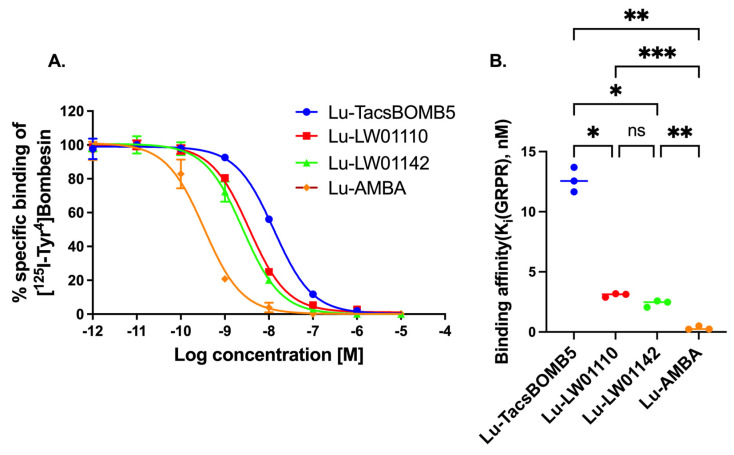
(**A**) Displacement curves of [^125^I-Tyr^4^]Bombesin by Lu-TacsBOMB5, Lu-LW01110, Lu-LW011142, and Lu-AMBA generated using GRPR-expressing prostate cancer PC-3 cells. (**B**) Comparison of the binding affinities of Lu-TacsBOMB5, Lu-LW01110, Lu-LW011142, and Lu-AMBA. ns, *, **, and *** indicate *p* > 0.05, <0.05, <0.01, and <0.001, respectively. Error bars indicate standard deviation (n = 3).

Lu-TacsBOMB5, Lu-LW01110, Lu-LW01142, and Lu-AMBA inhibited the binding of [^125^I-Tyr^4^]Bombesin to GRPR-expressing prostate cancer PC-3 cells in a dose-dependent manner (Figure 2A). The comparison of the binding affinity values (K_i_) for Lu-TacsBOMB5, Lu-LW01110, Lu-LW01142, and Lu-AMBA are shown in Figure 2B. Lu-AMBA showed the best binding affinity (K_i_ = 0.33 ± 0.16 nM) among all four Lu-complexed GRPR-targeted ligands. The binding affinities of Lu-LW01110 and Lu-LW01142 were comparable with K_i_ at a low nM scale (3.07 ± 0.15 vs. 2.37 ± 0.28 nM, *p* = 0.1462), and significantly better than that of Lu-TacsBOMB5 (12.6 ± 1.02 nM, *p* < 0.05)

**Figure 3:**
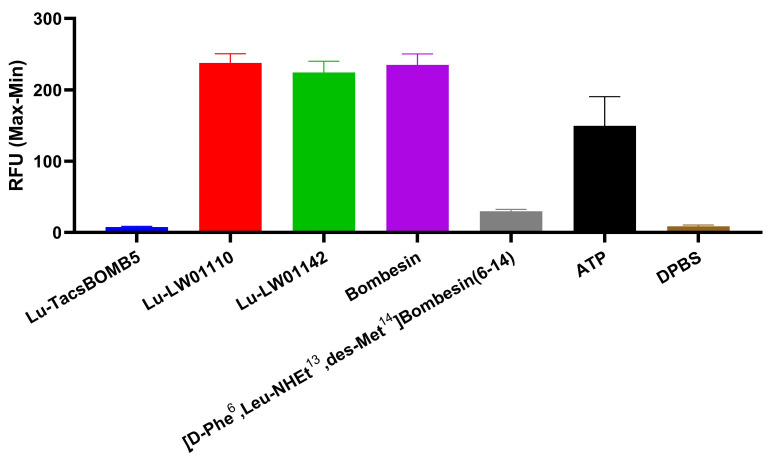
Intracellular calcium efflux in PC-3 cells induced by Lu-TacsBOMB5, Lu-LW01110, Lu-LW011142, bombesin, [D-Phe^6^,Leu-NHEt^13^,des-Met^14^]Bombesin(6–14), ATP, and DPBS. Error bars indicate standard deviation (n = 3).

The agonist/antagonist characterization for Lu-TacsBOMB5, Lu-LW01110, and Lu-LW011142 was determined via intracellular calcium release assay using PC-3 cells (Figure 3). Ca^2+^ efflux was induced by 50 nM of Lu-LW01110, Lu-LW01142, bombesin (agonist control), and ATP (positive control) with 238 ± 13.0, 224 ± 16.1, 235 ± 15.2, and 150 ± 40.7 RFUs, respectively, while 50 nM of Lu-TacsBOMB5, [D-Phe^6^,Leu-NHEt^13^,des-Met^14^]Bombesin(6–14) (antagonist control), and Dulbecco’s phosphate-buffered saline (DPBS, blank control) induced Ca^2+^ efflux corresponding to 7.56 ± 1.04, 29.7 ± 2.73, and 8.59 ± 1.88 RFUs, respectively. Based on the extents of induced calcium release, Lu-TacsBOMB5 was determined to be a GRPR antagonist, while Lu-LW01110 and Lu-LW01142 were determined to be GRPR agonists.

The hydrophilicity of [^177^Lu]Lu-TacsBOMB5, [^177^Lu]Lu-LW01110, and [^177^Lu]Lu-LW01142 was determined by the shake flask method following previously published procedures [21], and their logD_7.4_ values were −2.53 ± 0.20, −2.76 ± 0.10, and −2.28 ± 0.14, respectively (n = 3).

### 2.3. SPECT Imaging and Ex Vivo Biodistribution

**Figure 4:**
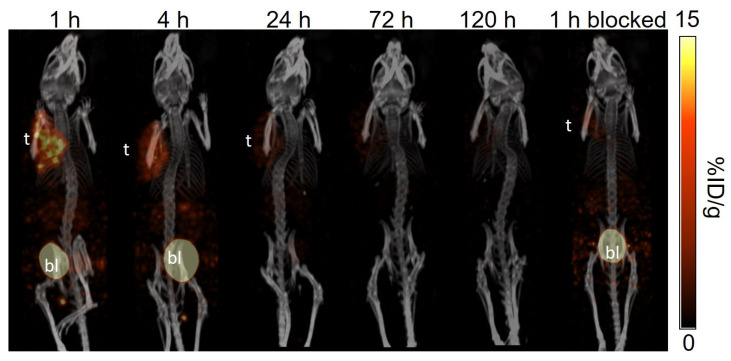
The representative longitudinal SPECT/CT images of [^177^Lu]Lu-TacsBOMB5 acquired from a PC-3 tumor-bearing NRG mouse at 1, 4, 24, 72, and 120 h post-injection. The blocked mouse was co-injected with 100 μg of [D-Phe^6^,Leu-NHEt^13^,des-Met^14^]Bombesin(6–14) and imaged at 1 h post-injection. t: tumor; bl: urinary bladder.

**Figure 5:**
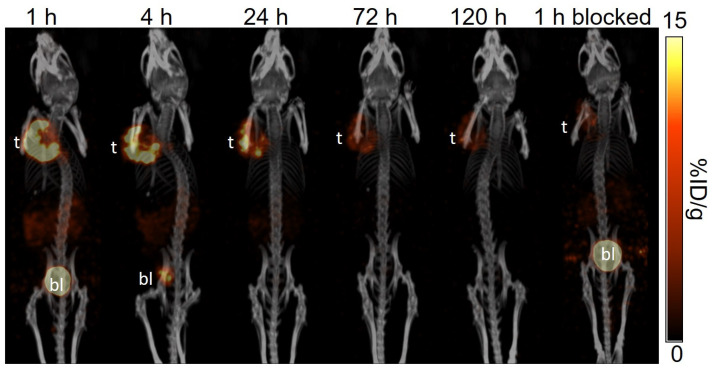
The representative longitudinal SPECT/CT images of [^177^Lu]Lu-LW01110 acquired from a PC-3 tumor-bearing NRG mouse at 1, 4, 24, 72, and 120 h post-injection. The blocked mouse was co-injected with 100 μg of [D-Phe^6^,Leu-NHEt^13^,des-Met^14^]Bombesin(6–14) and imaged at 1 h post-injection. t: tumor; bl: urinary bladder.

**Figure 6:**
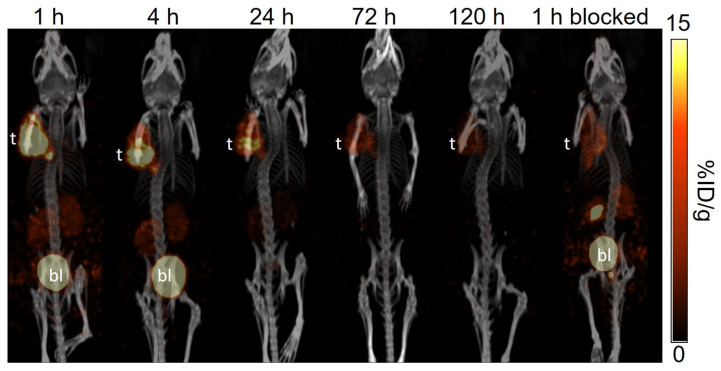
The representative longitudinal SPECT/CT images of [^177^Lu]Lu-LW01142 acquired from a PC-3 tumor-bearing NRG mouse at 1, 4, 24, 72, and 120 h post-injection. The blocked mouse was co-injected with 100 μg of [D-Phe^6^,Leu-NHEt^13^,des-Met^14^]Bombesin(6–14) and imaged at 1 h post-injection. t: tumor; bl: urinary bladder.

**Figure 7:**
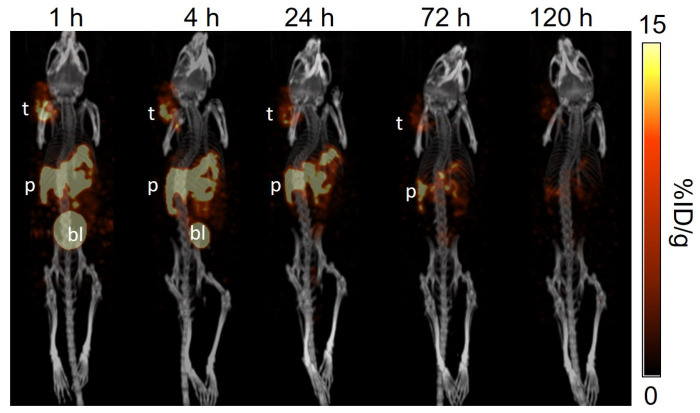
The representative longitudinal SPECT/CT images of [^177^Lu]Lu-AMBA acquired from a PC-3 tumor-bearing NRG mouse at 1, 4, 24, 72, and 120 h post-injection. t: tumor; p: pancreas; bl: urinary bladder.

The longitudinal SPECT/CT images of [^177^Lu]Lu-TacsBOMB5, [^177^Lu]Lu-LW01110, [^177^Lu]Lu-LW01142, and [^177^Lu]Lu-AMBA are shown in Figure 4, Figure 5, Figure 6 and Figure 7. [^177^Lu]Lu-TacsBOMB5, [^177^Lu]Lu-LW01110, and [^177^Lu]Lu-LW01142 were excreted mainly via the renal pathway with low radioactivity accumulated in normal organs/tissues. The urinary bladder was clearly visualized at 1 h and 4 h post-injection for all four radioligands. [^177^Lu]Lu-TacsBOMB5 enabled visualization of the PC-3 tumor xenografts in SPECT images at 1, 4, and 24 h post-injection (Figure 4). For [^177^Lu]Lu-LW01110 and [^177^Lu]Lu-LW01142, PC-3 tumor xenografts were visualized up to 120 h post-injection (Figure 5 and Figure 6). [^177^Lu]Lu-AMBA enabled visualization of the PC-3 tumor xenografts in the SPECT images up to 72 h post-injection (Figure 7). An extremely high accumulation of [^177^Lu]Lu-AMBA in the pancreas was observed up to 72 h post-injection (Figure 7). Co-injection with 100 μg of [D-Phe^6^,Leu-NHEt^13^,des-Met^14^]Bombesin(6–14) significantly decreased accumulation of [^177^Lu]Lu-TacsBOMB5, [^177^Lu]Lu-LW01110, and [^177^Lu]Lu-LW01142 in the PC-3 tumor xenografts at 1 h post-injection (Figure 4, Figure 5 and Figure 6).

**Figure 8:**
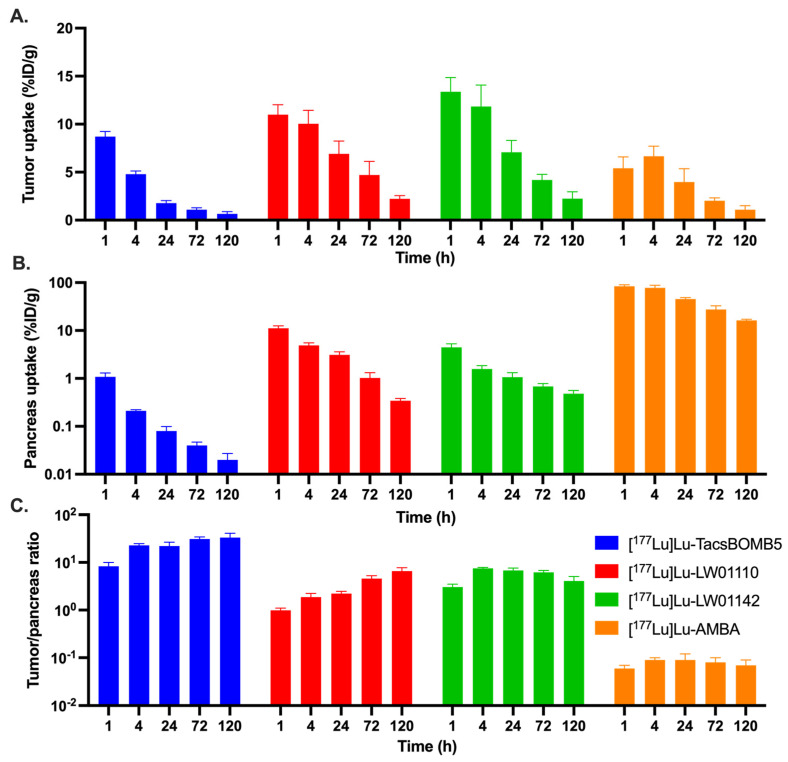
Comparison of [^177^Lu]Lu-TacsBOMB5, [^177^Lu]Lu-LW01110, [^177^Lu]Lu-LW01142, and [^177^Lu]Lu-AMBA on (**A**) their uptake in PC-3 tumor xenografts, (**B**) their uptake in the pancreas, and (**C**) the tumor/pancreas uptake ratios at 1, 4, 24, 72, and 120 h post-injection.

The ex vivo biodistribution results of [^177^Lu]Lu-TacsBOMB5, [^177^Lu]Lu-LW01110, [^177^Lu]Lu-LW01142, and [^177^Lu]Lu-AMBA are provided in Figure 8 and Tables S3–S6. All four ^177^Lu-labeled ligands showed moderate kidney accumulation, with the uptake values ranging from 3.47 to 7.70%ID/g at 1 h post-injection, which subsequently decreased to <1%ID/g after 72 h post-injection for [^177^Lu]Lu-TacsBOMB5, [^177^Lu]Lu-LW01110, and [^177^Lu]Lu-LW01142, and after 120 h post-injection for [^177^Lu]Lu-AMBA. The tumor uptake values of [^177^Lu]Lu-TacsBOMB5, [^177^Lu]Lu-LW01110, and [^177^Lu]Lu-LW01142 were 8.71 ± 0.53, 11.0 ± 1.03, and 13.4 ± 1.48%ID/g, respectively, at 1 h post-injection, and decreased over time. In contrast, the tumor uptake value of [^177^Lu]Lu-AMBA increased slightly from 5.42 ± 1.17%ID/g at 1 h post-injection to 6.66 ± 1.04%ID/g at 4 h post-injection, and then decreased afterward to 1.09 ± 0.42%ID/g at 120 h post-injection. A faster clearance from PC-3 tumor xenografts was observed for [^177^Lu]Lu-TacsBOMB5 compared with other GRPR-targeted ligands (Figure 8A and Tables S3–S6). The tumor uptake of [^177^Lu]Lu-TacsBOMB5 dropped to 1.77 ± 0.27%ID/g at 24 h post-injection and kept decreasing to < 1%D/g at 120 h post-injection (Figure 8A and Table S3). In contrast, the tumor uptake values of [^177^Lu]Lu-LW01110 and [^177^Lu]Lu-LW01142 were still >2%ID/g at 120 h post-injection (Figure 8A, Tables S4 and S5). The clinically evaluated GRPR agonist, [^177^Lu]Lu-AMBA, had significantly higher pancreas uptake than our GRPR-targeted ligands at all five time points, ranging from 83.8 ± 6.06%ID/g at 1 h post-injection to 16.3 ± 1.00%ID/g at 120 h post-injection (Figure 8B and Tables S3–S6). [^177^Lu]Lu-LW01110 had the second highest pancreas uptake with the uptake values of 11.1 ± 1.37, 4.91 ± 0.63, 3.10 ± 0.48, 1.03 ± 0.30, and 0.34 ± 0.04%ID/g at 1, 4, 24, 72 and 120 h post-injection, respectively. The other GRPR agonist, [^177^Lu]Lu-LW01142, showed significantly lower accumulation in the pancreas with only 4.45 ± 0.83%ID/g at 1 h post-injection, which decreased to around 1%ID/g at 24 h post-injection. Among all four ^177^Lu-labeled ligands, [^177^Lu]Lu-TacsBOMB5 showed the lowest accumulation in the pancreas, ranging from 1.08 ± 0.22%ID/g at 1 h post-injection to 0.02 ± 0.00%ID/g at 120 h post-injection. Furthermore, the accumulation of all three ^177^Lu-labeled ligands in most of collected organs/tissues were lower than that of [^177^Lu]Lu-AMBA, particularly in the intestines, stomach, and adrenal glands (Tables S3–S6).

The tumor/organ uptake ratios of [^177^Lu]Lu-TacsBOMB5, [^177^Lu]Lu-LW01110 and [^177^Lu]Lu-LW01142 were mostly increasing over time from 1 h to 72 h post-injection. With higher tumor uptake values, [^177^Lu]Lu-LW01110 and [^177^Lu]Lu-LW01142 had better tumor/blood and tumor/muscle uptake ratios than [^177^Lu]Lu-TacsBOMB5 over all five time-points. The average tumor/blood uptake ratios of [^177^Lu]Lu-TacsBOMB5 and [^177^Lu]Lu-LW01142 increased over time, rising from 4.95 ± 0.52 to 22.2 ± 6.11 for [^177^Lu]Lu-TacsBOMB5 and from 8.08 ± 0.92 to 1800 ± 724 for [^177^Lu]Lu-LW01142. The average tumor/blood uptake ratio of [^177^Lu]Lu-LW01110 increased over time from 15.9 ± 1.15 at 1 h post-injection, peaked at 72 h post-injection (1331 ± 177), but then decreased slightly to 1309 ± 412 at 120 h post-injection. With the lowest pancreas uptake values, [^177^Lu]Lu-TacsBOMB5 had the highest tumor/pancreas uptake ratios among all four evaluated ^177^Lu-labeled ligands ranging from 8.32 to 33.4 (Figure 8C and Tables S3–S6). On the other hand, [^177^Lu]Lu-AMBA showed the lowest tumor/pancreas uptake ratios at all time points among all four ligands (0.06 to 0.09, Figure 8C). The tumor/pancreas uptake ratios of [^177^Lu]Lu-LW01110 was 0.99 ± 0.11 at 1 h post-injection due to the comparable accumulation in PC-3 tumor xenograft and the pancreas, then subsequently increased to 6.60 ± 1.14 at 120 h post-injection.

Blocking studies of [^177^Lu]Lu-TacsBOMB5, [^177^Lu]Lu-LW01110, and [^177^Lu]Lu-LW01142 were conducted at 1 h post-injection (Tables S3–S5). The results showed that co-injection of [D-Phe^6^,Leu-NHEt^13^,des-Met^14^]Bombesin(6–14) reduced the average tumor uptake values of [^177^Lu]Lu-TacsBOMB5, [^177^Lu]Lu-LW01110, and [^177^Lu]Lu-LW01142 by 71%, 73%, and 62%, respectively. In addition, the average pancreas uptake values of [^177^Lu]Lu-TacsBOMB5, [^177^Lu]Lu-LW01110, and [^177^Lu]Lu-LW01142 were also reduced by 45%, 76% and 58%, respectively, for the blocked mice. Significant reductions were also observed on the uptake values of [^177^Lu]Lu-TacsBOMB5, [^177^Lu]Lu-LW01110, and [^177^Lu]Lu-LW01142 for the intestines and stomach (Tables S3–S5).

### 2.4. Radiation Dosimetry

**Figure 9:**
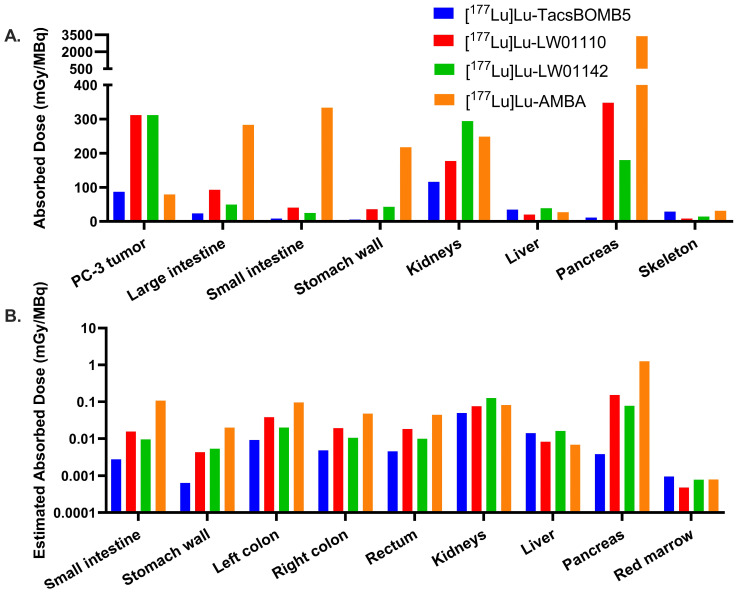
(**A**) Comparison of the radiation absorbed doses for [^177^Lu]Lu-TacsBOMB5, [^177^Lu]Lu-LW01110, [^177^Lu]Lu-LW01142, and [^177^Lu]Lu-AMBA in the PC-3 tumor xenograft and the organs of interest in mice; (**B**) comparison of the estimated radiation absorbed doses for [^177^Lu]Lu-TacsBOMB5, [^177^Lu]Lu-LW01110, [^177^Lu]Lu-LW01142, and [^177^Lu]Lu-AMBA in organs of interest in adult human males.

The calculated radiation absorbed doses in mice from [^177^Lu]Lu-TacsBOMB5, [^177^Lu]Lu-LW01110, [^177^Lu]Lu-LW01142, and [^177^Lu]Lu-AMBA are presented in Figure 9A and Table S7. The absorbed doses of [^177^Lu]Lu-LW01110 and [^177^Lu]Lu-LW01142 in a 1 g PC-3 tumor xenograft (Unit Density Sphere Model) were calculated to be the same (312 mGy/MBq), higher than that of [^177^Lu]Lu-TacsBOMB5 (87.1 mGy/MBq) and [^177^Lu]Lu-AMBA (79.1 mGy/MBq). For [^177^Lu]Lu-TacsBOMB5, [^177^Lu]Lu-LW01110, and [^177^Lu]Lu-LW01142, the urinary bladder received the highest radiation absorbed dose among all the target organs with 562, 1260, and 1180 mGy/MBq, respectively. For [^177^Lu]Lu-AMBA, the pancreas received the highest absorbed dose (3380 mGy/MBq), followed by the urinary bladder (1300 mGy/MBq). The absorbed doses of all four evaluated ^177^Lu-labeled ligands in kidneys were relatively high, ranging from 116 to 294 mGy/MBq. Except [^177^Lu]Lu-AMBA, [^177^Lu]Lu-LW01110 showed the highest absorbed dose in the pancreas (348 mGy/MBq), which was nearly two-fold of that for [^177^Lu]Lu-LW01142 (180 mGy/MBq) and approximately 30-fold of that for [^177^Lu]Lu-TacsBOMB5 (11.6 mGy/MBq). [^177^Lu]Lu-AMBA also had the highest absorbed doses in some major organs including the large intestine, small intestine, and stomach wall, while [^177^Lu]Lu-TacsBOMB5 had the lowest absorbed doses in these major organs.

By extrapolating the mouse ex vivo biodistribution data to the adult human male model, the estimated radiation absorbed doses for all four radioligands were calculated and provided in Figure 9B and Table S8. The estimated absorbed dose for [^177^Lu]Lu-AMBA (1.25 × 10^0^ mGy/MBq) in the pancreas was approximately 8-fold higher than that of [^177^Lu]Lu-LW01110 (1.53 × 10^−1^ mGy/MBq) and 16-fold higher than that of [^177^Lu]Lu-LW01142 (7.79 × 10^−2^ mGy/MBq). [^177^Lu]Lu-TacsBOMB5 showed the lowest estimated absorbed dose in the pancreas (3.83 × 10^−3^ mGy/MBq), which was only 0.3% of that of [^177^Lu]Lu-AMBA. In addition, the estimated absorbed doses of [^177^Lu]Lu-AMBA in most selected organs/tissues were higher than our novel ^177^Lu-labeled ligands, including the esophagus, intestines, and stomach. The effective whole-body doses of [^177^Lu]Lu-TacsBOMB5, [^177^Lu]Lu-LW01110, [^177^Lu]Lu-LW01142, and [^177^Lu]Lu-AMBA to an adult human male were 6.49 × 10^−3^, 1.35 × 10^−2^, 1.16 × 10^−2^, and 3.13 × 10^−2^ mSv/MBq, respectively.

## 3. Discussion

GRPR is a very promising target for cancer diagnosis and therapy [1,2,3,4,5]. Previously, our group reported the synthesis and evaluation of three GRPR-targeted tracers derived from the [Thz^14^]Bombesin(6–14) sequence, including a GRPR antagonist ([^68^Ga]Ga-TacsBOMB5) and two GRPR agonists ([^68^Ga]Ga-LW01110 and [^68^Ga]Ga-LW01142) [16,17]. These three tracers showed high uptake in PC-3 tumor xenografts and minimal pancreas uptake at 1 h post-injection, demonstrating their potential as imaging agents for detecting GRPR-expressing lesions with PET [16,17]. Therefore, we replaced ^68^Ga in these three promising tracers with ^177^Lu and evaluated the potential of the resulting ligands as radiotherapeutic agents.

Lu-LW01110, Lu-LW01142, and Lu-ABMA showed excellent binding affinities toward GRPR with K_i_ values ranging from 0.33 to 3.07 nM, while the binding affinity of Lu-TacsBOMB5 (K_i_ = 12.6 nM) was significantly inferior to that of Lu-LW01110, Lu-LW01142, and Lu-ABMA (Figure 2). A significant decrease was observed in the GRPR binding affinity for Lu-TacsBOMB5 and Lu-LW01110 in comparison with their Ga-complexed analogs: K_i_ = 6.09 ± 0.95 nM for Ga-TacsBOMB5 vs. 12.6 ± 1.02 nM for Lu-TacsBOMB5, *p* = 0.0013; K_i_ = 1.39 ± 0.03 nM for Ga-LW01110 vs. 3.07 ± 0.15 nM for Lu-LW01110, *p* < 0.001 [16,17]. For LW01142, no significant difference was observed between its Lu- and Ga-complexed analogs (2.37 ± 0.28 vs. 3.19 ± 0.78 nM) [17].

The antagonist characteristics of Lu-TacsBOMB5 and the agonist characteristics of Lu-LW01110 and Lu-LW01142 were confirmed by intracellular calcium release assay (Figure 3). The results are consistent with the antagonist/agonist characteristics of Ga-complexed TacsBOMB5, LW01110, and LW01142 reported previously [16,17]. These data indicate that replacing Ga with Lu retains the original antagonist/agonist characteristics. The hydrophilicity nature of [^177^Lu]Lu-TacsBOMB5, [^177^Lu]Lu-LW01110, and [^177^Lu]Lu-LW01142 was confirmed by their low logD_7.4_ values ranging from −2.28 to −2.76, which contributed to their predominantly renal excretion as shown in the SPECT images presented in Figure 4, Figure 5 and Figure 6.

Similar to the observations of [^68^Ga]Ga-TacsBOMB5, [^68^Ga]Ga-LW01110, and [^68^Ga]Ga-LW01142 on the PET images [16,17], [^177^Lu]Lu-TacsBOMB5, [^177^Lu]Lu-LW01110, and [^177^Lu]Lu-LW01142 all had minimal uptake in the pancreas and enabled clear visualization of the PC-3 tumor xenografts in SPECT images (Figure 4, Figure 5 and Figure 6) with good tumor-to-background contrast at 1 h post-injection. On the contrary, the pancreas was clearly visualized in the SPECT images by [^177^Lu]Lu-AMBA up to 72 h post-injection (Figure 7).

A faster clearance of [^177^Lu]Lu-TacsBOMB5 from PC-3 tumor xenografts was observed from the SPECT/CT images in comparison with the two novel GRPR agonists ([^177^Lu]Lu-LW01110 and [^177^Lu]Lu-LW01142) and [^177^Lu]Lu-AMBA. Consistent with the observation from the SPECT/CT images, the tumor retention of [^177^Lu]Lu-TacsBOMB5 was relatively shorter when compared with [^177^Lu]Lu-LW01110, [^177^Lu]Lu-LW01142, and [^177^Lu]Lu-AMBA. Approximately 80% uptake of [^177^Lu]Lu-TacsBOMB5 in PC-3 tumor xenografts was cleared within 24 h, compared with 37% of [^177^Lu]Lu-LW01110, 47% of [^177^Lu]Lu-LW01142, and 27% of [^177^Lu]Lu-AMBA. Furthermore, the tumor uptake of [^177^Lu]Lu-TacsBOMB5 was lower than that of [^177^Lu]Lu-LW01110 and [^177^Lu]Lu-LW01142 at 1 h post-injection. This might be partly due to the lowest binding affinity of Lu-TacsBOMB5 among all four radioligands (Figure 2), in addition to its being an antagonist which is not internalized upon binding to the receptor.

The tumor uptake values of [^177^Lu]Lu-TacsBOMB5 (8.71 ± 0.53%ID/g) and [^177^Lu]Lu-LW01110 (11.0 ± 1.03%ID/g) at 1 h post-injection were lower than that of previously reported [^68^Ga]Ga-TacsBOMB5 (15.7 ± 2.17%ID/g) and [^68^Ga]Ga-LW01110 (16.6 ± 1.60%ID/g), respectively, using the same tumor model. This might be partly due to the inferior binding affinities (K_i_) of Lu-TacsBOMB5 (12.6 ± 1.02 nM) and Lu-LW01110 (3.07 ± 0.15 nM) compared to their Ga-complexed analogs, Ga-TacsBOMB5 (6.09 ± 0.95 nM) and Ga-LW01110 (1.39 ± 0.03 nM) [16,17].

[^177^Lu]Lu-AMBA showed an extremely high uptake in the pancreas (83.8 ± 6.06%ID/g at 1 h post-injection), along with significant accumulation in the intestines and stomach across all five time points. This pattern aligns with the reported physiological expression of GRPR in normal organs and tissues [1]. The much higher uptake of [^177^Lu]Lu-AMBA in the pancreas than that in the PC-3 tumor xenograft might be due to its more selective binding to the mouse GRPR expressed on the mouse pancreas than the human GRPR expressed on PC-3 tumor xenografts. However, this hypothesis needs to be further verified in the future. The pancreas uptake of [^177^Lu]Lu-TacsBOMB5, [^177^Lu]Lu-LW01110, and [^177^Lu]Lu-LW01142 at early time points are much lower than most of reported ^177^Lu-labeled GRPR-targeted ligands, including [^177^Lu]Lu-RM2, [^177^Lu]Lu-AMTG, and [^177^Lu]Lu-NeoBOMB1 [22,23,24]. This suggests that replacing ^68^Ga in our previously reported ^68^Ga-labeled [Thz^14^]Bombesin(6–14) derivatives with ^177^Lu retains their minimal pancreas uptake characteristics [16,17]. Owing to the rapid clearance from the blood and muscle, the tumor/blood and tumor/muscle uptake ratios of all four radioligands increased over time. The better tumor uptake of [^177^Lu]Lu-LW01110 and [^177^Lu]Lu-LW01142 compared with the GRPR antagonist [^177^Lu]Lu-TacsBOMB5 also contributed to their better tumor/blood and tumor/muscles uptake ratios. [^177^Lu]Lu-TacsBOMB5, [^177^Lu]Lu-LW01110, and [^177^Lu]Lu-LW01142 showed significantly higher tumor/pancreas ratios at all five time-points compared with [^177^Lu]Lu-AMBA, suggesting better biodistribution profiles and lower off-target uptake of our GRPR-targeted ligands.

The significantly reduced tumor uptake of [^177^Lu]Lu-TacsBOMB5, [^177^Lu]Lu-LW01110, and [^177^Lu]Lu-LW01142 in the blocked mice demonstrates their GRPR-targeting specificity (Figure 4, Figure 5 and Figure 6 and Tables S3–S5). The significant uptake reductions of [^177^Lu]Lu-TacsBOMB5, [^177^Lu]Lu-LW01110, and [^177^Lu]Lu-LW01142 in the pancreas, intestines, and stomach are also in agreement with the physiological expression pattern of GRPR in normal organs/tissues, particularly for the pancreas which is the normal organ with the highest physiological GRPR expression [1].

Corroborated with observations from the SPECT/CT images, the high calculated radiation absorbed doses received by the urinary bladders and kidneys for [^177^Lu]Lu-TacsBOMB5, [^177^Lu]Lu-LW01110, [^177^Lu]Lu-LW01142, and [^177^Lu]Lu-AMBA indicate that the renal pathway is the main excretion pathway for all four radioligands. With the lowest initial uptake in PC-3 tumor xenografts among all evaluated radioligands, the clinically evaluated GRPR agonist [^177^Lu]Lu-AMBA also had the lowest absorbed dose in PC-3 tumor xenografts (79.1 mGy/MBq). On the other hand, [^177^Lu]Lu-AMBA showed the highest absorbed dose to the pancreas (3380 mGy/MBq) due to its extremely high pancreas uptake. Compared to [^177^Lu]Lu-LW01110 and [^177^Lu]Lu-LW01142 (312 mGy/MBq), [^177^Lu]Lu-TacsBOMB5 had less radiation absorbed dose to PC-3 tumor xenografts (87.1 mGy/MBq). This is due to the lower tumor uptake and faster tumor clearance of [^1^⁷⁷Lu]Lu-TacsBOMB5, likely resulting from its lower binding affinity and antagonist characteristics—lack of internalization upon binding to the receptor.

Compared with the previously reported preclinical dosimetry data of [^177^Lu]Lu-RM2, the radiation absorbed doses of [^177^Lu]Lu-LW01110 (312 mGy/MBq), [^177^Lu]Lu-LW01142 (312 mGy/MBq), and [^177^Lu]Lu-TacsBOMB5 (87.1 mGy/MBq) to PC-3 tumor xenografts were lower than that of [^177^Lu]Lu-RM2 (429 mGy/MBq) [22]. One possible explanation is their relatively shorter tumor retention due to their inferior binding affinities compared to that of Lu-RM2 (1.19 ± 0.16 nM). The reported radiation absorbed dose of [^177^Lu]Lu-RM2 in the mouse pancreas is 316 mGy/MBq, which is around 1.8-fold higher than that of [^177^Lu]Lu-LW01142 (180 mGy/MBq) and >27-fold higher than that of [^177^Lu]Lu-TacsBOMB5 (11.6 mGy/MBq), but slight lower than that of [^177^Lu]Lu-LW01110 (348 mGy/MBq). With the lowest absorbed dose in the pancreas, [^177^Lu]Lu-TacsBOMB5 had the highest tumor/pancreas absorbed dose ratio (6.22), followed by [^177^Lu]Lu-LW01142 (1.47) and [^177^Lu]Lu-RM2 (1.36) [22]. Resulting from the higher absorbed dose in the pancreas compared with PC-3 tumor xenografts, the tumor/pancreas absorbed dose ratios of [^177^Lu]Lu-LW01110 and [^177^Lu]Lu-AMBA were lower than 1, with a ratio of 0.80 and 0.02, respectively. Therefore, future development of GRPR-targeted radiotherapeutic agents should focus on not only improving tumor absorbed dose to enhance treatment efficacy, but also increasing the tumor/pancreas absorbed dose ratio to minimize potential toxicity to the pancreas.

In agreement with the mouse dosimetry data, the extrapolated human dosimetry data (Figure 9B and Table S8) also indicates that the pancreas is the main dose-limiting organ of GRPR-targeted radiopharmaceuticals [11,25]. The pancreas showed the highest estimated absorbed doses for [^177^Lu]Lu-LW01110 (1.53 × 10^−1^ mGy/MBq), [^177^Lu]Lu-LW01142 (7.79 × 10^−2^ mGy/MBq), and [^177^Lu]Lu-AMBA (1.25 mGy/MBq) among all the selected organs and tissues except the kidneys and urinary bladder, due to their renal excretion. In contrast, the GRPR antagonist [^177^Lu]Lu-TacsBOMB5 showed the lowest estimated absorbed dose in the pancreas (3.83 × 10^−3^ mGy/MBq), among all four radioligands. Compared with the published dosimetry data of the clinically validated GRPR antagonists, [^177^Lu]Lu-RM2 and [^177^Lu]Lu-NeoB (previously named NeoBOMB1), both [^177^Lu]Lu-LW01142 and [^177^Lu]Lu-TacsBOMB5 showed lower estimated absorbed doses in the pancreas. The estimated absorbed dose of [^177^Lu]Lu-LW01142 in the pancreas is 67% of that of [^177^Lu]Lu-RM2 (1.16 × 10^−1^ mGy/MBq), and only 28% of that of [^177^Lu]Lu-NeoB (2.82 × 10^−1^ mGy/MBq) [22,24]. The GRPR antagonist [^177^Lu]Lu-TacsBOMB5 had a slightly higher estimated absorbed dose to the pancreas than our previously published Pro^14^ analog [^177^Lu]Lu-ProBOMB5 (1.98 × 10^−3^ mGy/MBq), but had only 1.4–1.9% of the estimated absorbed doses of [^177^Lu]Lu-RM2 and [^177^Lu]Lu-NeoB in the pancreas [22,24]. Moreover, lower estimated absorbed doses of [^177^Lu]Lu-TacsBOMB5 and [^177^Lu]Lu-LW01142 were observed in other GRPR-expressing organs/tissues, such as the stomach wall, small intestine, colon, and rectum, compared to that of [^177^Lu]Lu-RM2 and [^177^Lu]Lu-NeoB [22,24]. These data suggest that both [^177^Lu]Lu-TacsBOMB5 and [^177^Lu]Lu-LW01142 can reduce the radiation exposure to some normal organ/tissues, particularly to the pancreas. Mouse-to-human extrapolation assumes similar pharmacokinetics between these two species. However, species differences in GRPR expression may lead to an overestimation or underestimation of the pancreas doses.

The relatively shorter tumor retention and lower overall absorbed doses delivered to tumors might limit their therapeutic applications and further optimizations are needed for both [^177^Lu]Lu-TacsBOMB5 and [^177^Lu]Lu-LW01142 to enhance treatment efficacy. One potential design strategy is to improve the binding affinity toward GRPR. Obeid et al. recently reported that replacing the PEG2 linker in a GRPR-targeted tracer, [^111^In]In-AU-RM26-M2, with a positively charged linker, Arg-Arg, resulted in [^111^In]In-AU-RM26-M4 with a greatly improved binding affinity [26]. The K_D_1 and K_D_2 values were 315 and 1680 pM, respectively, for [^111^In]In-AU-RM26-M2 and 5.8 and 6.0 pM, respectively, for [^111^In]In-AU-RM26-M4. The greatly improved GRPR binding affinity also led to a higher uptake in PC-3 tumor xenografts at 4 h pi (15%ID/g for [^111^In]In-AU-RM26-M4 vs. 7%ID/g for [^111^In]In-AU-RM26-M2). Therefore, the addition of Arg-Arg between the DOTA chelator and our GRPR-targeted sequences could be a promising strategy to improve their GRPR binding affinity. Another strategy is to extend the blood residence time of the radioligand to maximize tumor uptake and enhance treatment efficacy. Introduction of an albumin-binding motif is widely used in the design of radiotherapeutic agents to improve tumor uptake and increase the radiation absorbed dose by prolonging the blood residence time [27,28]. However, this strategy is more suitable for radioligands with extremely high in vivo stability in plasma, as prolonged circulation of radiometabolites would increase radiation absorbed doses to normal organs/tissues, potentially leading to undesired toxicities.

## 4. Materials and Methods

### 4.1. General Methods

TacsBOMB5, LW01110, LW01142, and AMBA were synthesized following published procedures [16,17,29]. All the other chemicals and solvents were sourced commercially and used without further purification. Purification and quality control of ^nat^Lu/^177^Lu-complexed ligands were conducted on Agilent (Santa Clara, CA, USA) HPLC systems equipped with a model 1200 quaternary pump, a model 1200 UV absorbance detector (220 nm), and a Bioscan (Washington, DC, USA) NaI scintillation detector. The operation of the Agilent HPLC systems was controlled by the Agilent ChemStation software (version A.01.05 (1.3.19.115)). The purification and quality control were conducted using a semi-preparative column (Luna C18, 5 µm, 250 × 10 mm) and an analytical column (Luna C18, 5 µm, 250 × 4.6 mm), respectively, purchased from Phenomenex (Torrance, CA, USA). The HPLC eluates containing the desired products were collected and lyophilized with a Labconco (Kansas City, MO, USA) FreeZone 4.5 Plus freeze-drier. MS analyses of the nonradioactive Lu-complexed standards were performed with a Waters (Milford, MA, USA) Acquity QDa mass spectrometer equipped with a 2489 UV/Vis detector and an e2695 Separations module. C18 Sep-Pak cartridges (1 cm^3^, 50 mg) were purchased from Waters (Milford, MA, USA). The ^177^LuCl_3_ was purchased from Isotopia Molecular Imaging Ltd. (Petah Tikva, Israel) and ITM Medical Isotopes GmbH (Munich, Germany). The radioactivity of ^177^Lu-labeled peptides was measured using a Capintec (Ramsey, NJ, USA) CRC^®^-25R/W dose calibrator, and the radioactivity of samples collected from biodistribution studies, binding assays, and logD_7.4_ assays were measured using a Perkin Elmer (Waltham, MA, USA) Wizard2 2480 automatic gamma counter or a Hidex (Turku, Finland) automatic gamma counter. Calcium release assays were performed on a FlexStation 3 microplate reader (Molecular Devices, San Jose, CA, USA). Internal dosimetry calculations were performed using the organ level internal dose assessment software (OLINDA, version 2.2.3, Hermes Medical Solutions, Stockholm, Sweden).

### 4.2. Synthesis of ^177^Lu-Labeled Compounds

Lutetium-177 radiolabeling for all four ligands was conducted following previously reported procedures [21]. Briefly, 843–1043 MBq of ^177^LuCl_3_ solution was added into a 4 mL glass vial containing 0.7 mL of NaOAc buffer (0.1 M, pH 4.5) and 10 μL precursor solution (1 mM), followed by a 15 min incubation at 95 °C. The ^177^Lu-labeled compounds were separated from uncomplexed ^177^Lu and precursors by HPLC using a semi-preparative column. The eluate fraction containing the radiolabeled product was collected, diluted with water (50 mL), and passed through a C18 Sep-Pak cartridge pre-washed with 10 mL of ethanol and 10 mL of water. The ^177^Lu-labeled product trapped on the cartridge was eluted off with ethanol (0.4 mL), and diluted with 1 mL of Dulbecco’s phosphate buffered saline (DPBS) for SPECT imaging and ex vivo biodistribution studies. Quality control was conducted using HPLC with an analytical column. The HPLC conditions for purification and quality control are provided in Table S2.

### 4.3. The LogD_7.4_ Measurement

The logD_7.4_ values of all the ^177^Lu-labeled GRPR-targeted ligands were measured using the shake flask method as previously reported [21]. An aliquot (~2 MBq) of the radiolabeled product was added to a mixture of n-octanol (3 mL) and DPBS (3 mL, pH 7.4), followed by 1 min vortexing and 15 min centrifugation at 5000 rpm. Samples of the n-octanol (1 mL) and buffer (1 mL) layers were collected, and the activity was counted using a gamma counter. The logD_7.4_ values were calculated using the following equation:

$${logD}_{7.4}={log}_{10}[\frac{{\mathrm{counts}}_{n-\mathrm{octanol}\mathrm{phase}}}{{\mathrm{counts}}_{\mathrm{butter}\mathrm{phase}}}]$$

### 4.4. In Vitro Competition Binding Assay

The in vitro competition binding assay was conducted following a previously reported method [21]. PC-3 cells were seeded at 2 × 10^5^ cells/well in 24-well poly-D-lysine plates 48 h prior to the assay. The growth medium was replaced with 400 μL of reaction medium (RPMI 1640 containing 2 mg/mL bovine serum albumin (BSA), and 20 mM HEPES). After 1 h incubation, 50 μL/well of nonradioactive Lu-complexed standard at decreasing concentrations (10 μM to 1 pM), and 50 μL/well of 0.01 nM [^125^I-Tyr^4^]Bombesin were added. The plates were incubated at 37 °C with gentle agitation for 1 h. After that, the cells were washed with ice-cold DPBS twice gently, and harvested by trypsinization. Radioactivity from each well was counted using a gamma counter, and the data were analyzed using nonlinear regression (one binding site model for competition assay) with GraphPad (San Diego, CA, USA) Prism 10 software (Version 10.1.1). Nonspecific binding was assessed in the presence of 10 μM of the tested Lu-complexed ligand.

### 4.5. Fluorometric Calcium Release Assay

The antagonist/agonist characteristics of Lu-TacsBOMB5, Lu-LW01110, and Lu-LW01142 were determined following previously published procedure [21,30]. A 96-well clear bottom black plate was seeded with 5 × 10^4^ PC-3 prostate cancer cells/100 μL/well 24 h prior to the assay. The calcium-sensitive dye (FLIPR Calcium 6 assay kit from Molecular Devices, San Jose, CA, USA) in loading buffer (100 μL/well) was added into the 96-well plate, followed by 1 h incubation at 37 °C. After that, the plate was transferred into a FlexStation 3 microplate reader (Molecular Devices, San Jose, CA, USA). Lu-complexed nonradioactive standard (50 nM), [D-Phe^6^,Leu-NHEt^13^,des-Met^14^]Bombesin(6–14) (50 nM, antagonist control), bombesin (50 nM, agonist control), adenosine triphosphate (ATP, 50 nM, positive control), or DPBS (blank control) was added, and the fluorescent signals were acquired for 2 min (λ_Ex_ = 485 nm; λ_Em_ = 525 nm; n = 3). The relative fluorescent unit (RFU = max − min) was calculated to determine the agonistic/antagonistic characteristics for the tested ligands.

### 4.6. SPECT/CT Imaging and Ex Vivo Biodistribution Studies

SPECT/CT imaging experiments were performed using an MILabs (Houten, The Netherlands) U-SPECT-II/CT scanner with a custom-made ultra-high-sensitivity big mouse collimator (2 mm pinhole size) following previously reported procedures [21]. The experiments were conducted according to the guidelines established by the Canadian Council on Animal Care and approved by Animal Ethics Committee of the University of British Columbia (protocol number A20-0113, approved on 30 September 2022). Male NOD.Cg-Rag1^tm1Mom^ Il2rg^tm1Wjl^/SzJ (NRG) mice were used in these experiments because of their immunodeficient characteristics and excellent radiation tolerance. Mice were anaesthetized and subcutaneously implanted with 5 × 10^6^ PC-3 prostate cancer cells (100 µL; 1:1 PBS/Matrigel) behind the left shoulder. SPECT/CT imaging and biodistribution studies were performed once the tumors reached a diameter of 5–8 mm, typically after approximately 4 weeks of growth.

The PC-3 tumor-bearing mice were sedated (2.5% isoflurane in O_2_) and injected with ^177^Lu-labeled ligands (15–32 MBq) through a lateral caudal tail vein. The mice were imaged at 1, 4, 24, 72, and 120 h post-injection. At each time point, a 5 min CT scan was obtained using 615 μA and 60 kV parameters for localization and attenuation, followed by 2 × 30 min static SPECT scans acquired in list mode with an energy window centered around 208 keV. The MILabs U-SPECT II reconstruction software (version 8.00) was used to reconstruct data, and the images were decay corrected to the time of injection with PMOD v 3.402 (PMOD Technologies GmbH, Fallanden, Switzerland).

For ex vivo biodistribution studies, the mice were injected with ~2–6 MBq of the radioligand. At 1, 4, 24, 72, and 120 h post-injection, the mice were anesthetized with 2% isoflurane and euthanized by CO_2_ inhalation. Blocking study was conducted at 1 h post-injection via co-injection of the radioligand with 100 μg of [D-Phe^6^,Leu-NHEt^13^,des-Met^14^]Bombesin(6–14). Organs/tissues of interested were collected, weighed, and measured using an automatic gamma counter. Uptake values were expressed as the percentage of the injected dose per gram of tissue (%ID/g).

### 4.7. Dosimetry Analysis

The uptake values (%ID/g) obtained from the ex vivo biodistribution data (n = 5) were decayed to the appropriate time point and fitted to mono- or bi-exponential equations using SciPy library [31] integrated into an in-house Python script (Python Software Foundation v.3.10.12). The best fit was selected based on maximizing the coefficient of determination (R^2^) and minimizing the residuals. Time–activity curves calculated from the parameters obtained from the best fit for each organ were then integrated and normalized to injected activity to acquire time-integrated activity coefficients (TIACs) per unit gram, and subsequently multiplied by the mass of model tissue (25 g or 30 g mouse phantom). The TIACs were corrected for the tumor sink effect following the formula adopted in the report by Cicone et al. as shown below [32]:

$${TIAC}_{m,corrected}\left(organ\right)={TIAC}_{m}\left(organ\right)+\left\lceil {TIAC}_{m}\left(tumor\right)\times \frac{{TIAC}_{m}\left(organ\right)}{{TIAC}_{m}\left(WB\right){-TIAC}_{m}\left(tumor\right)}\right\rceil$$

The TIAC values were input in OLINDA (Hermes Medical Solutions, v2.2.3) [33] which has pre-calculated dose factors for mouse models [34]. The mouse biodistribution data were extrapolated to humans using the method proposed by Kirschner et al. using the following equation [35]:

$${TIAC}_{H}\left(organ\right)={TIAC}_{M}\left(organ\right)\times \left\lceil \frac{{m(organ)}_{H}/{WB}_{H}}{{m(organ)}_{M}/{WB}_{M}}\right\rceil$$

where m(organ)_H_ and m(organ)_M_ are masses of human and mouse organs, respectively, and *WB* represents total-body mass. The human TIACs calculated using the above equation were input into OLINDA and dosimetry results were assessed for the ICRP 89 Adult Male Model [36]. The %ID/g value for the blood was assumed to be that for the heart contents of the phantom. Finally, the TIAC for the tumor was also calculated based on the biodistribution data, and the values were input into the sphere model available in OLINDA [37].

### 4.8. Statistical Analysis

Statistical analyses were performed via Student’s *t*-test using the Microsoft (Redmond, WA, USA) Excel software (version 16.84 (24041420)) or two-way ANOVA tests using the GraphPad (San Diego, CA, USA) Prism 10 software (Version 10.1.1). The unpaired two-tailed test was used to compare ex vivo biodistribution data between two ligands. The unpaired one-tailed test was used to compare ex vivo the biodistribution data of unblocked and blocked groups. Two-way ANOVA was used to compare the binding affinities for nonradioactive Lu-complexed standards. Statistically significant difference was considered when the adjusted *p* value was <0.05 (* *p* < 0.05, ** *p* < 0.01, and *** *p* < 0.001).

## 5. Conclusions

In this study, we synthesized one GRPR antagonist ([^177^Lu]Lu-TacsBOMB5) and two GRPR agonist ([^177^Lu]Lu-LW01110 and [^177^Lu]Lu-LW01142) radioligands and performed longitudinal SPECT/CT imaging and biodistribution studies and dosimetry calculations. All three Lu-complexed ligands retained the same antagonist/agonist characteristics as their Ga-complexed analogs, demonstrating that substitution of the radiometal in these radioligands does not change their antagonist/agonist characteristics. Consistent with the low pancreas uptake of their ^68^Ga-labeled analogs observed from our previous studies, [^177^Lu]Lu-TacsBOMB5, [^177^Lu]Lu-LW01110, and [^177^Lu]Lu-LW01142 all showed significantly lower pancreas uptake than the clinically evaluated [^177^Lu]Lu-AMBA at all five time points. However, the longer retention of [^177^Lu]Lu-LW01110 in the pancreas gave rise to a higher radiation absorbed dose delivered to the pancreas compared to that of [^177^Lu]Lu-RM2 [22]. In contrast, both [^177^Lu]Lu-TacsBOMB5 and [^177^Lu]Lu-LW01142 showed lower absorbed doses in the pancreas and some other key organs suggesting that both radioligands have better safety profiles. However, relatively faster clearance of [^177^Lu]Lu-TacsBOMB5 and [^177^Lu]Lu-LW01142 from the PC-3 tumor xenografts gave rise to lower radiation absorbed doses delivered to tumors. Thus, further optimizations are required to improve tumor uptake and prolong tumor retention of both [^177^Lu]Lu-TacsBOMB5 and [^177^Lu]Lu-LW01142 to enhance treatment efficacy.

## 6. Patents

The compounds disclosed in this report are covered by pending patent application(s), in which Lei Wang, Kuo-Shyan Lin, and François Bénard are listed as inventors.

## Data Availability

The original contributions presented in the study are included in the article/Supplementary Materials, further inquiries can be directed to the corresponding author.

## Supplementary Materials

The following supporting information can be downloaded at: https://www.mdpi.com/article/10.3390/ph18040449/s1. Table S1: MS characterizations, yields, and HPLC conditions for purification and quality control of Lu-TacsBOMB5, Lu-LW01110, Lu-LW01142, and Lu-AMBA; Table S2: HPLC conditions for purification and quality control of ^177^Lu-labeled TacsBOMB5, LW01110, LW01142, and AMBA; Figure S1: The representative MS spectrum of Lu-TacsBOMB5; Figure S2: The representative MS spectrum of Lu-LW01110; Figure S3: The representative MS spectrum of Lu-LW01142; Figure S4: The representative MS spectrum of Lu-AMBA; Table S3: Biodistribution and tumor/organ uptake ratios of [^177^Lu]Lu-TacsBOMB5 in PC-3 tumor-bearing mice at 1, 4, 24, 72, and 120 h post-injection; Table S4: Biodistribution and tumor/organ uptake ratios of [^177^Lu]Lu-LW01110 in PC-3 tumor-bearing mice at 1, 4, 24, 72, and 120 h post-injection; Table S5: Biodistribution and tumor/organ uptake ratios of [^177^Lu]Lu-LW01142 in PC-3 tumor-bearing mice at 1, 4, 24, 72, and 120 h post-injection; Table S6: Biodistribution and tumor/organ uptake ratios of [^177^Lu]Lu-AMBA in PC-3 tumor-bearing mice at 1, 4, 24, 72, and 120 h post-injection; Table S7: Estimated radiation absorbed doses (per unit of injected radioactivity (mGy/MBq)) in mice for [^177^Lu]Lu-TacsBOMB5, [^177^Lu]Lu-LW01110, [^177^Lu]Lu-LW01142, and [^177^Lu]Lu-AMBA; Table S8: Estimated radiation absorbed doses (mGy/MBq) in adult human males for [^177^Lu]Lu-TacsBOMB5, [^177^Lu]Lu-LW01110, [^177^Lu]Lu-LW01142, and [^177^Lu]Lu-AMBA.
