# Supplementary material for: Novel 177Lu-Labeled [Thz14]Bombesin(6–14) Derivatives with Low Pancreas Accumulation for Targeting Gastrin-Releasing Peptide Receptor-Expressing Cancer
Source: Pharmaceuticals (Basel). 2025 Mar 23;18(4):449. doi: 10.3390/ph18040449 (PMC12030706; doi:10.3390/ph18040449)
Supplement: Supplementary file 1 [file pharmaceuticals-18-00449-s001.zip › pharmaceuticals-3540589-supplementary.pdf]

Supporting Information

Novel ^{177}Lu-labeled [Thz^{14}]Bombesin(6-14) derivatives with low pancreas accumulation for targeting gastrin-releasing peptide receptor-expressing cancer

Lei Wang [1], Devon E. Chapple [1], Hsiou-Ting Kuo [1], Sara Kurkowska [2,3], Ryan P. Wilson [1], Wing Sum Lau [1], Pauline Ng [1], Carlos Uribe [2,4,5], François Bénard [1,4,5], Kuo-Shyan Lin [1,4,5*]

[1] *Department of Molecular Oncology, BC Cancer Research Institute, Vancouver, BC V5Z 1L3, Canada*

[2] *Department of Integrative Oncology, BC Cancer Research Institute, Vancouver, BC V5Z 1L3, Canada*

[3] *Department of Nuclear Medicine, Pomeranian Medical University, Szczecin 70-204, Poland*

[4] *Department of Molecular Imaging and Therapy, BC Cancer, Vancouver, BC V5Z 4E6, Canada*

[5] *Department of Radiology, University of British Columbia, Vancouver, BC V5Z 1M9, Canada*

*** Email: klin@bccrc.ca**

Synthesis of Nonradioactive Lu-complexed Standards

The nonradioactive Lu-complexed standards were synthesized following previously reported method by mixing and incubating the precursor solution with $LuCl_3$ (10 eq.) in NaOAc buffer (0.1 M, 500 µL, pH 4.5) at 90 °C for 30 min [1]. The reaction mixture was then purified via HPLC (semi-preparative column, flow rate: 4.5 mL/min). The HPLC eluates containing the desired peptides were collected and lyophilized. The HPLC conditions, isolated yields and MS confirmations of the nonradioactive Lu-complexed standards are provided in Table S1 and Figures S1-S4.

Table S1: MS characterizations, yields and HPLC conditions for purification and quality control of Lu-TacsBOMB5, Lu-LW01110, Lu-LW01142, and Lu-AMBA.

Compound name	HPLC conditions		Retention time (min)	Yield (%)	Calculated mass (m/z)	Found (m/z)
Lu-TacsBOMB5	Semi-Prep	25% CH_3CN and 0.1% TFA in H_2O; flow rate 4.5 mL/min	13.9	47	$[M+2H]^{2+}$ 885.4	$[M+2H]^{2+}$ 885.6
	QC	26% CH_3CN and 0.1% TFA in H_2O; flow rate 2.0 mL/min	8.7	/		
Lu-LW01110	Semi-Prep	24% CH_3CN and 0.1% TFA in H_2O; flow rate 4.5 mL/min	18.0	88	$[M+2H]^{2+}$ 899.4	$[M+2H]^{2+}$ 899.6
	QC	24% CH_3CN and 0.1% TFA in H_2O; flow rate 2.0 mL/min	11.0	/		
Lu-LW01142	Semi-Prep	24% CH_3CN and 0.1% TFA in H_2O; flow rate 2.0 mL/min	12.6	61	$[M+2H]^{2+}$ 903.9	$[M+2H]^{2+}$ 904.1
	QC	24% CH_3CN and 0.1% TFA in H_2O; flow rate 2.0 mL/min	7.2	/		
Lu-AMBA	Semi-Prep	24% CH_3CN and 0.1% TFA in H_2O; flow rate 4.5 mL/min	11.5	45	$[M+2H]^{2+}$ 837.9	$[M+2H]^{2+}$ 837.9
	QC	24% CH_3CN and 0.1% TFA in H_2O; flow rate 2.0 mL/min	7.6	/		

Table S2: HPLC conditions for purification and quality control of ^{177}Lu-labeled TacsBOMB5, LW01110, LW01142, and AMBA. FA: formic acid.

Compound name	HPLC conditions		Retention time (min)
[^{177}Lu]Lu-TacsBOMB5	Semi-Prep	22% CH_3CN and 0.1% FA in H_2O; flow rate 4.5 mL/min	9.5
	QC	24% CH_3CN and 0.1% FA in H_2O; flow rate 2 mL/min	6.1
[^{177}Lu]Lu-LW01110	Semi-Prep	19% CH_3CN and 0.1% FA in H_2O; flow rate 4.5 mL/min	16.1
	QC	22% CH_3CN and 0.1% FA in H_2O; flow rate 2 mL/min	6.8
[^{177}Lu]Lu-LW01142	Semi-Prep	16% CH_3CN and 0.1% FA in H_2O; flow rate 4.5 mL/min	10.9
	QC	19% CH_3CN and 0.1% FA in H_2O; flow rate 2.0 mL/min	7.0
[^{177}Lu]Lu-AMBA	Semi-Prep	21% CH_3CN and 0.1% FA in H_2O; flow rate 4.5 mL/min	7.0
	QC	22% CH_3CN and 0.1% FA in H_2O; flow rate 2.0 mL/min	9.4

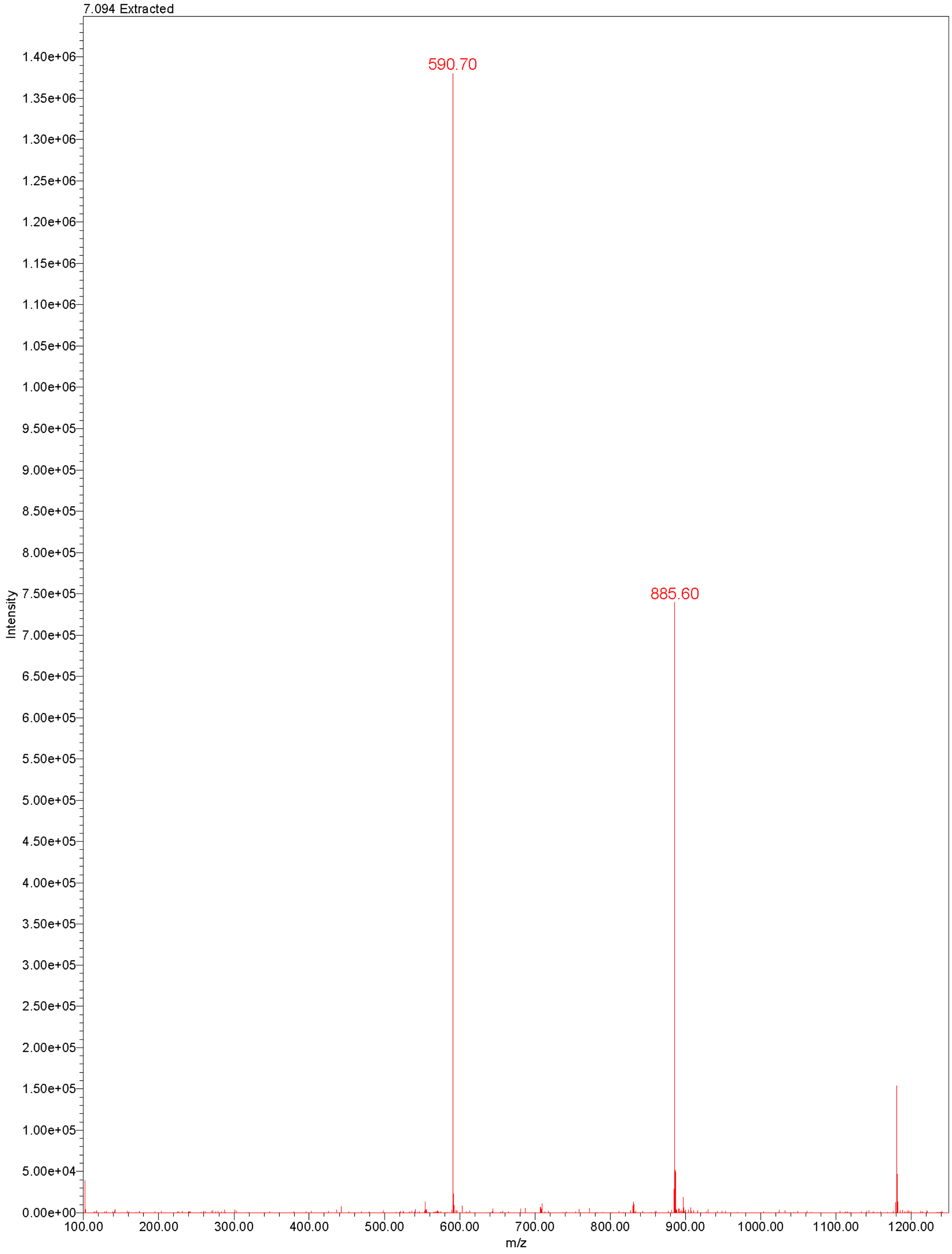

Figure S1: The representative MS spectrum of Lu-TacsBOMB5: calculated $[M+2H]^{2+}$ (m/z) 855.4; found 855.6.

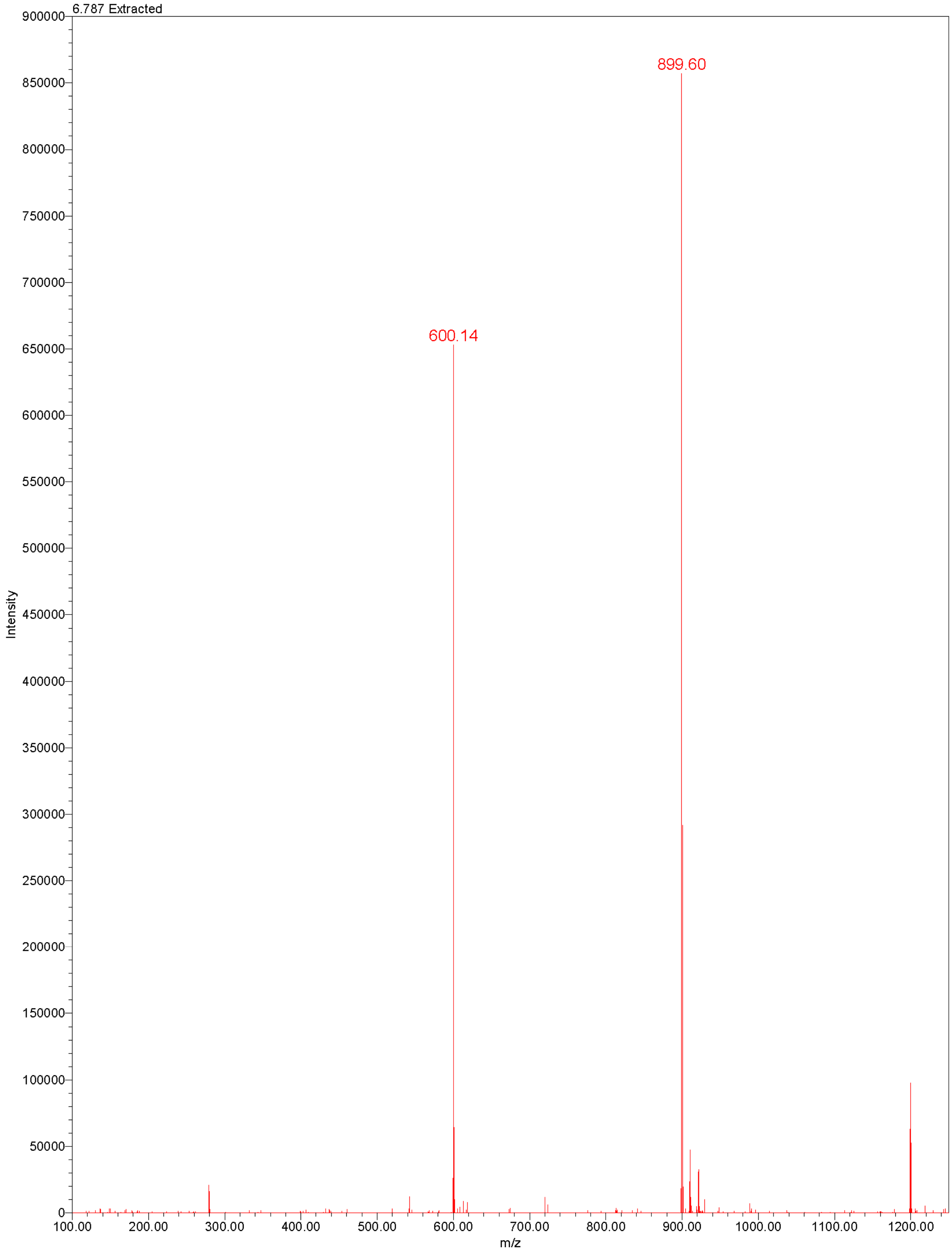

Figure S2: The representative MS spectrum of Lu-LW01110: calculated $[M+2H]^{2+}$ (m/z) 899.4; found 899.6.

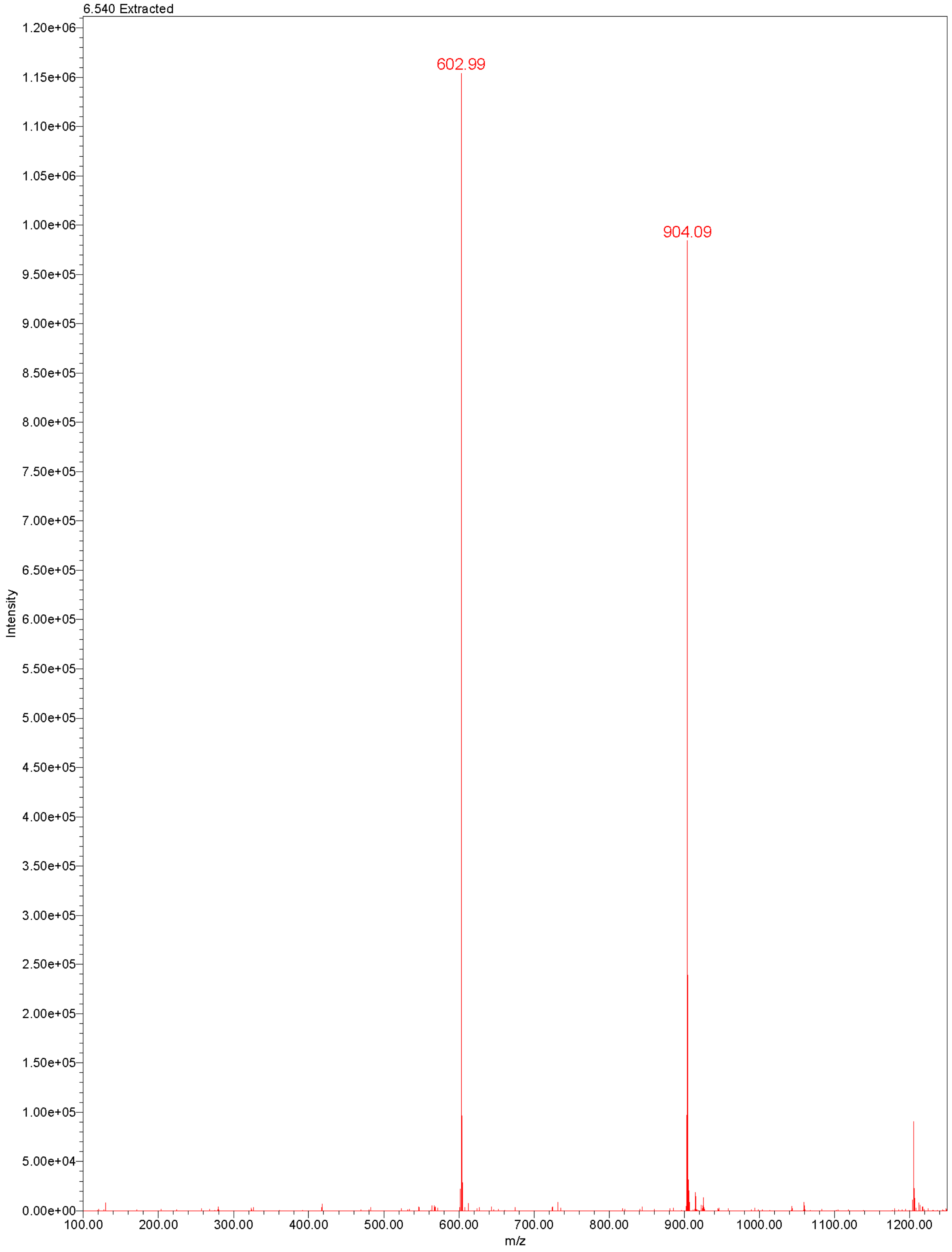

Figure S3: The representative MS spectrum of Lu-LW01142: calculated $[M+2H]^{2+}$ (m/z) 903.9; found 904.1.

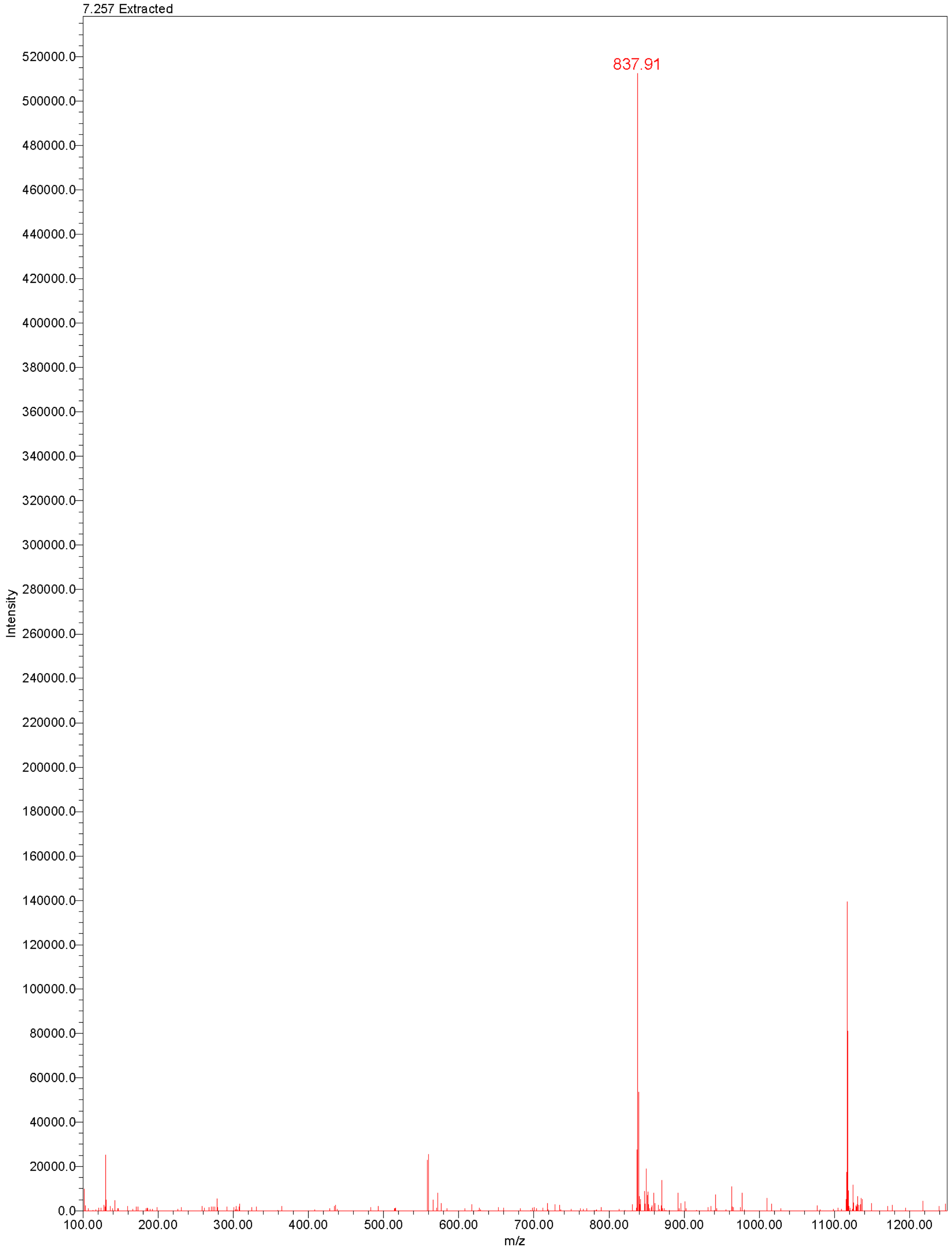

Figure S4: The representative MS spectrum of Lu-AMBA: calculated $[M+2H]^{2+}$ (m/z) 837.9; found 837.9.

Cell Culture

The PC-3 prostate adenocarcinoma cell line was obtained from ATCC (via Cedarlane, Burlington, Canada), and verified pathogen-free via IMPACT Rodent Pathogen Test (IDEXX BioAnalytics, Columbia, MO, USA). The cells were cultured using RPMI 1640 medium (Life Technologies, Carlsbad, CA, USA) containing 10% FBS, penicillin (100 U/mL) and streptomycin (100 μg/mL) at 37 °C in a Panasonic Healthcare (Tokyo, Japan) MCO-19AIC humidified incubator containing 5% CO_2. The cells were washed with DPBS and harvested following a 1-min trypsinization when grown to 80-90% confluence at passages 16-20 to ensure consistent GRPR expression. The cell concentration was counted in triplicate using a Moxi mini automated cell counter (ORFLO Technologies, Ketchum, ID, USA).

Table S3: Biodistribution (mean ± SD, n = 5) and tumor-to-organ uptake ratios of [^{177}Lu]Lu-TacsBOMB5 in PC-3 tumor-bearing mice at 1, 4, 24, 72, and 120 h post-injection. The mice in the blocked group were co-injected with 100 μg of [D-Phe^{6},Leu-NHEt^{13},des-Met^{14}]Bombesin(6-14) per mouse. *, **, and *** indicate p < 0.05, < 0.01 and < 0.001, respectively, when comparing the 1 h and 1 h blocked data of [^{177}Lu]Lu-TacsBOMB5.

Tissue (%ID/g, n = 5)	[^{177}Lu]Lu-TacsBOMB5					
	1 h	4 h	24 h	72 h	120 h	1 h blocked
Blood	1.77 ± 0.14	0.85 ± 0.03	0.21 ± 0.02	0.07 ± 0.01	0.03 ± 0.01	0.45 ± 0.11***
Fat	0.15 ± 0.04	0.06 ± 0.01	0.03 ± 0.01	0.02 ± 0.01	0.01 ± 0.01	0.06 ± 0.01***
Testes	0.45 ± 0.11	0.23 ± 0.04	0.12 ± 0.02	0.08 ± 0.04	0.08 ± 0.02	0.18 ± 0.02***
Small intestine	0.60 ± 0.16	0.19 ± 0.05	0.09 ± 0.03	0.03 ± 0.00	0.02 ± 0.00	0.26 ± 0.04**
Large intestine	0.31 ± 0.09	0.42 ± 0.09	0.42 ± 0.25	0.10 ± 0.08	0.03 ± 0.02	0.13 ± 0.04**
Spleen	0.31 ± 0.03	0.23 ± 0.04	0.16 ± 0.03	0.18 ± 0.05	0.14 ± 0.06	0.15 ± 0.02***
**Pancreas**	**1.08 ± 0.22**	**0.21 ± 0.01**	**0.08 ± 0.02**	**0.04 ± 0.01**	**0.02 ± 0.00**	**0.59 ± 0.11****
Stomach	0.24 ± 0.06	0.11 ± 0.05	0.27 ± 0.16	0.03 ± 0.00	0.02 ± 0.02	0.06 ± 0.01***
Liver	0.67 ± 0.07	0.54 ± 0.04	0.29 ± 0.02	0.20 ± 0.02	0.16 ± 0.07	0.29 ± 0.05***
Adrenal glands	0.61 ± 0.28	0.29 ± 0.07	0.17 ± 0.02	0.18 ± 0.12	0.12 ± 0.08	0.17 ± 0.04*
Kidneys	3.47 ± 0.29	3.10 ± 0.35	1.38 ± 0.18	0.60 ± 0.07	0.39 ± 0.11	2.78 ± 0.51*
Heart	0.45 ± 0.06	0.21 ± 0.03	0.09 ± 0.01	0.05 ± 0.01	0.04 ± 0.01	0.15 ± 0.02***
Lungs	2.20 ± 0.93	1.45 ± 0.34	0.25 ± 0.03	0.13 ± 0.01	0.07 ± 0.03	0.37 ± 0.04**
**PC-3 tumor**	**8.71 ± 0.53**	**4.80 ± 0.32**	**1.77 ± 0.27**	**1.09 ± 0.19**	**0.66 ± 0.23**	**2.53 ± 0.34*****
Bone	0.12 ± 0.02	0.09 ± 0.03	0.07 ± 0.02	0.07 ± 0.01	0.07 ± 0.01	0.06 ± 0.03**
Muscle	0.22 ± 0.01	0.10 ± 0.02	0.03 ± 0.00	0.02 ± 0.00	0.01 ± 0.00	0.13 ± 0.03***
Brain	0.04 ± 0.00	0.02 ± 0.00	0.00 ± 0.00	0.00 ± 0.00	0.00 ± 0.00	0.02 ± 0.00***
Tumor-to-organ Uptake Ratios						
Tumor/muscle	39.6 ± 2.10	49.9 ± 8.90	51.5 ± 6.89	53.6 ± 7.29	48.2 ± 20.3	20.6 ± 3.63
Tumor/blood	4.95 ± 0.52	5.64 ± 0.30	8.34 ± 1.81	15.5 ± 3.40	22.2 ± 6.11	5.88 ± 1.56***
Tumor/kidney	2.52 ± 0.27	1.56 ± 0.18	1.29 ± 0.19	1.80 ± 0.23	1.70 ± 0.32	0.94 ± 0.21**
Tumor/pancreas	8.32 ± 1.70	22.8 ± 2.08	22.2 ± 4.34	31.0 ± 3.31	33.4 ± 7.57	4.42 ± 0.82***

Table S4: Biodistribution (mean ± SD, n = 5) and tumor-to-organ uptake ratios of [^{177}Lu]Lu-LW01110 in PC-3 tumor-bearing mice at 1, 4, 24, 72, and 120 h post-injection. The mice in the blocked group were co-injected with 100 µg of [D-Phe^{6},Leu-NHEt^{13},des-Met^{14}]Bombesin(6-14) per mouse. *, **, and *** indicate $p < 0.05$, < 0.01 and < 0.001, respectively, when comparing the 1 h and 1 h blocked data of [^{177}Lu]Lu-LW01110.

Tissue (%ID/g, n = 5)	[^{177}Lu]Lu-LW01110					
	1 h	4 h	24 h	72 h	120 h	1 h blocked
Blood	0.69 ± 0.06	0.04 ± 0.01	0.01 ± 0.00	0.00 ± 0.00	0.00 ± 0.00	0.58 ± 0.16
Fat	0.07 ± 0.02	0.01 ± 0.00	0.01 ± 0.00	0.00 ± 0.00	0.00 ± 0.00	0.07 ± 0.02
Testes	0.23 ± 0.04	0.06 ± 0.05	0.02 ± 0.01	0.02 ± 0.00	0.01 ± 0.00	0.30 ± 0.16
Small intestine	2.01 ± 0.13	0.56 ± 0.20	0.30 ± 0.10	0.09 ± 0.03	0.03 ± 0.00	0.49 ± 0.14***
Large intestine	1.00 ± 0.15	1.15 ± 0.38	0.44 ± 0.11	0.23 ± 0.04	0.09 ± 0.02	0.29 ± 0.08***
Spleen	0.28 ± 0.05	0.15 ± 0.03	0.12 ± 0.02	0.10 ± 0.02	0.05 ± 0.01	0.21 ± 0.05
**Pancreas**	**11.1 ± 1.37**	**4.91 ± 0.63**	**3.10 ± 0.48**	**1.03 ± 0.30**	**0.34 ± 0.04**	**2.71 ± 0.76*** **
Stomach	0.99 ± 0.21	0.43 ± 0.16	0.17 ± 0.07	0.08 ± 0.02	0.04 ± 0.02	0.17 ± 0.10***
Liver	0.40 ± 0.05	0.23 ± 0.07	0.13 ± 0.04	0.07 ± 0.01	0.04 ± 0.01	0.26 ± 0.05**
Adrenal glands	1.22 ± 0.38	1.02 ± 0.33	0.98 ± 0.18	0.41 ± 0.06	0.25 ± 0.05	0.54 ± 0.24**
Kidneys	3.77 ± 0.58	2.67 ± 0.49	1.46 ± 0.42	0.52 ± 0.10	0.20 ± 0.02	3.56 ± 0.77
Heart	0.22 ± 0.01	0.06 ± 0.06	0.03 ± 0.00	0.02 ± 0.00	0.01 ± 0.00	0.20 ± 0.06
Lungs	0.69 ± 0.12	0.23 ± 0.11	0.07 ± 0.03	0.06 ± 0.03	0.03 ± 0.03	0.52 ± 0.08*
**PC-3 tumor**	**11.0 ± 1.03**	**10.1 ± 1.40**	**6.90 ± 1.34**	**4.72 ± 1.42**	**2.23 ± 0.33**	**2.99 ± 0.42*** **
Bone	0.14 ± 0.05	0.05 ± 0.03	0.02 ± 0.01	0.02 ± 0.01	0.01 ± 0.00	0.08 ± 0.02
Muscle	0.15 ± 0.02	0.03 ± 0.01	0.01 ± 0.00	0.01 ± 0.00	0.00 ± 0.00	0.14 ± 0.03
Brain	0.03 ± 0.00	0.03 ± 0.02	0.00 ± 0.00	0.00 ± 0.00	0.00 ± 0.00	0.02 ± 0.00**
Tumor-to-organ Uptake Ratios						
Tumor/muscle	76.2 ± 10.1	392 ± 101	651 ± 95.0	647 ± 311	776 ± 314	21.5 ± 3.27***
Tumor/blood	15.9 ± 1.55	234 ± 53.9	1218 ± 229	1331 ± 177	1309 ± 412	5.32 ± 0.87***
Tumor/kidney	2.96 ± 0.43	3.27 ± 0.89	4.86 ± 0.64	8.92 ± 1.62	11.3 ± 1.27	0.85 ± 0.10***
Tumor/pancreas	0.99 ± 0.11	1.87 ± 0.37	2.22 ± 0.25	4.58 ± 0.68	6.60 ± 1.14	1.17 ± 0.31

Table S5: Biodistribution (mean ± SD, n = 5) and tumor-to-organ uptake ratios of [^{177}Lu]Lu-LW01142 in PC-3 tumor-bearing mice at 1, 4, 24, 72, and 120 h post-injection. The mice in the blocked group were co-injected with 100 µg of [D-Phe^{6},Leu-NHEt^{13},des-Met^{14}]Bombesin(6-14) per mouse. *, **, and *** indicate $p < 0.05$, < 0.01 and < 0.001, respectively, when comparing the 1 h and 1 h blocked data of [^{177}Lu]Lu-LW01142.

Tissue (%ID/g, n = 5)	[^{177}Lu]Lu-LW01142					
	1 h	4 h	24 h	72 h	120 h	1 h blocked
Blood	1.67 ± 0.21	0.08 ± 0.01	0.01 ± 0.00	0.00 ± 0.00	0.00 ± 0.00	1.34 ± 0.35
Fat	0.11 ± 0.04	0.02 ± 0.01	0.01 ± 0.00	0.01 ± 0.00	0.00 ± 0.00	0.14 ± 0.04
Testes	0.32 ± 0.18	0.05 ± 0.01	0.03 ± 0.01	0.02 ± 0.00	0.01 ± 0.00	0.48 ± 0.16
Small intestine	1.32 ± 0.42	0.29 ± 0.08	0.14 ± 0.03	0.05 ± 0.01	0.02 ± 0.02	0.54 ± 0.10**
Large intestine	0.76 ± 0.16	0.81 ± 0.21	0.30 ± 0.12	0.13 ± 0.05	0.07 ± 0.04	0.31 ± 0.08***
Spleen	0.47 ± 0.07	0.17 ± 0.04	0.14 ± 0.05	0.09 ± 0.02	0.09 ± 0.02	0.40 ± 0.04
**Pancreas**	**4.45 ± 0.83**	**1.57 ± 0.28**	**1.06 ± 0.26**	**0.68 ± 0.10**	**0.48 ± 0.08**	**1.88 ± 0.42*** **
Stomach	0.60 ± 0.24	0.11 ± 0.04	0.16 ± 0.07	0.10 ± 0.05	0.02 ± 0.01	0.17 ± 0.06**
Liver	0.71 ± 0.08	0.33 ± 0.05	0.28 ± 0.07	0.12 ± 0.03	0.08 ± 0.02	0.57 ± 0.15
Adrenal glands	1.43 ± 0.50	0.59 ± 0.06	0.41 ± 0.08	0.20 ± 0.05	0.17 ± 0.09	0.75 ± 0.33
Kidneys	6.86 ± 0.81	5.51 ± 0.54	2.64 ± 0.94	0.60 ± 0.17	0.30 ± 0.07	7.44 ± 1.71
Heart	0.45 ± 0.06	0.06 ± 0.01	0.02 ± 0.01	0.02 ± 0.00	0.01 ± 0.00	0.43 ± 0.14
Lungs	1.53 ± 0.19	0.57 ± 0.21	0.16 ± 0.08	0.03 ± 0.01	0.03 ± 0.01	1.06 ± 0.27*
**PC-3 tumor**	**13.4 ± 1.48**	**11.8 ± 2.24**	**7.07 ± 1.23**	**4.18 ± 0.6**	**2.25 ± 0.71**	**5.03 ± 0.43*** **
Bone	0.15 ± 0.03	0.03 ± 0.01	0.03 ± 0.01	0.03 ± 0.01	0.02 ± 0.01	0.13 ± 0.03
Muscle	0.22 ± 0.04	0.03 ± 0.01	0.01 ± 0.00	0.01 ± 0.00	0.00 ± 0.00	0.23 ± 0.11
Brain	0.03 ± 0.01	0.01 ± 0.00	0.00 ± 0.00	0.00 ± 0.00	0.00 ± 0.00	0.03 ± 0.01
Tumor-to-organ Uptake Ratios						
Tumor/muscle	60.1 ± 5.97	454 ± 53.3	672 ± 161	736 ± 189	588 ± 92.8	25.2 ± 10.1***
Tumor/blood	8.08 ± 0.92	162 ± 52.0	841 ± 162	1620 ± 500	1800 ± 724	3.91 ± 0.87***
Tumor/kidney	1.95 ± 0.04	2.14 ± 0.26	2.88 ± 0.84	7.34 ± 1.83	7.46 ± 1.11	0.70 ± 0.15***
Tumor/pancreas	3.06 ± 0.45	7.53 ± 0.35	6.78 ± 0.88	6.18 ± 0.60	4.08 ± 1.00	2.79 ± 0.69

Table S6: Biodistribution (mean ± SD, n = 5) and tumor-to-organ uptake ratios of [^{177}Lu]Lu-AMBA in PC-3 tumor-bearing mice at 1, 4, 24, 72, and 120 h post-injection.

Tissue (%ID/g, n = 5)	[^{177}Lu]Lu-AMBA				
	1 h	4 h	24 h	72 h	120 h
Blood	0.65 ± 0.17	0.05 ± 0.01	0.02 ± 0.00	0.01 ± 0.00	0.00 ± 0.00
Fat	0.08 ± 0.04	0.02 ± 0.01	0.01 ± 0.00	0.01 ± 0.01	0.00 ± 0.00
Testes	0.26 ± 0.05	0.06 ± 0.01	0.03 ± 0.00	0.02 ± 0.00	0.01 ± 0.00
Small intestine	8.42 ± 0.56	8.40 ± 0.98	4.59 ± 0.44	2.39 ± 0.71	1.19 ± 0.23
Large intestine	5.41 ± 0.52	7.81 ± 1.09	4.69 ± 0.43	1.57 ± 0.35	0.86 ± 0.39
Spleen	0.80 ± 0.35	0.63 ± 0.24	0.40 ± 0.15	0.17 ± 0.05	0.11 ± 0.02
**Pancreas**	**83.8 ± 6.06**	**77.7 ± 10.5**	**45.4 ± 3.30**	**27.6 ± 5.37**	**16.3 ± 1.00**
Stomach	3.23 ± 2.32	2.39 ± 0.65	2.36 ± 0.77	0.88 ± 0.25	0.44 ± 0.10
Liver	0.46 ± 0.10	0.75 ± 0.65	0.22 ± 0.06	0.09 ± 0.03	0.05 ± 0.01
Adrenal glands	20.4 ± 3.91	31.2 ± 6.49	12.5 ± 1.35	2.78 ± 0.87	0.70 ± 0.06
Kidneys	7.70 ± 0.76	7.01 ± 1.27	2.91 ± 0.08	1.76 ± 0.28	0.87 ± 0.13
Heart	0.27 ± 0.08	0.08 ± 0.01	0.04 ± 0.00	0.02 ± 0.00	0.01 ± 0.00
Lungs	0.72 ± 0.14	0.28 ± 0.03	0.14 ± 0.03	0.06 ± 0.02	0.03 ± 0.01
**PC-3 tumor**	**5.42 ± 1.17**	**6.66 ± 1.04**	**3.97 ± 1.39**	**2.03 ± 0.30**	**1.09 ± 0.42**
Bone	0.47 ± 0.04	0.37 ± 0.08	0.21 ± 0.01	0.12 ± 0.02	0.07 ± 0.03
Muscle	0.27 ± 0.02	0.12 ± 0.03	0.05 ± 0.01	0.02 ± 0.00	0.01 ± 0.00
Brain	0.05 ± 0.01	0.05 ± 0.01	0.04 ± 0.01	0.02 ± 0.01	0.02 ± 0.00
Tumor-to-organ Uptake Ratios					
Tumor/muscle	20.4 ± 4.03	58.6 ± 15.4	72.5 ± 22.5	88.9 ± 15.6	103 ± 46.0
Tumor/blood	8.86 ± 3.05	135 ± 22.4	180 ± 64.3	264 ± 53.9	391 ± 157
Tumor/kidney	0.70 ± 0.14	0.96 ± 0.10	1.26 ± 0.47	1.16 ± 0.12	1.24 ± 0.42
Tumor/pancreas	0.06 ± 0.01	0.09 ± 0.01	0.09 ± 0.03	0.08 ± 0.02	0.07 ± 0.02

Table S7: Estimated radiation absorbed doses (per unit of injected radioactivity (mGy/MBq)) in mice for [^{177}Lu]Lu-TacsBOMB5, [^{177}Lu]Lu-LW01110, [^{177}Lu]Lu-LW01142, and [^{177}Lu]Lu-AMBA.

Target Organ	[^{177}Lu]Lu-TacsBOMB5	[^{177}Lu]Lu-LW01110	[^{177}Lu]Lu-LW01142	[^{177}Lu]Lu-AMBA
PC-3 tumor	87.1	312	312	79.1
Brain	2.52	1.79	3.7	4.74
Large intestine	23.6	92.9	49.5	283
Small intestine	8.58	40.4	25	334
Stomach wall	5.49	35.8	42.7	218
Heart	12.2	2.71	4.65	5.32
Kidneys	116	177	294	249
Liver	34.4	20.2	38.8	27
Lungs	29	19.1	22.7	12.1
Pancreas	11.6	348	180	3380
Skeleton	28.6	8.3	14.5	31.3
Spleen	33.8	27.4	35.4	75
Testes	18.2	15.4	4.46	5.03
Thyroid	2.22	0.87	1.65	1.98
Bladder	562	1260	1180	1300
Total body	11.5	20.9	20	89.4

Table S8: Estimated radiation absorbed doses (mGy/MBq) in adult human males for [^{177}Lu]Lu-TacsBOMB5, [^{177}Lu]Lu-LW01110, [^{177}Lu]Lu-LW01142, and [^{177}Lu]Lu-AMBA.

Target Organ	[^{177}Lu]Lu-TacsBOMB5	[^{177}Lu]Lu-LW01110	[^{177}Lu]Lu-LW01142	[^{177}Lu]Lu-AMBA
Adrenals	1.17E-02	6.13E-02	2.82E-02	2.31E-01
Brain	1.02E-04	4.10E-04	9.04E-04	1.08E-03
Esophagus	4.89E-04	5.08E-04	7.08E-04	1.41E-03
Eyes	1.87E-04	1.97E-04	3.53E-04	3.70E-04
Gallbladder wall	7.53E-04	8.12E-04	1.16E-03	1.97E-03
Left colon	9.17E-03	3.82E-02	2.02E-02	9.57E-02
Small intestine	2.80E-03	1.56E-02	9.57E-03	1.07E-01
Stomach wall	6.36E-04	4.30E-03	5.38E-03	1.99E-02
Right colon	4.83E-03	1.94E-02	1.05E-02	4.79E-02
Rectum	4.58E-03	1.83E-02	9.97E-03	4.41E-02
Heart	8.30E-03	1.63E-03	3.12E-03	2.81E-03
Kidneys	4.97E-02	7.57E-02	1.27E-01	8.12E-02
Liver	1.42E-02	8.32E-03	1.63E-02	6.91E-03
Lungs	1.29E-02	9.27E-03	1.03E-02	4.10E-03
Pancreas	3.83E-03	1.53E-01	7.79E-02	1.25E+00
Prostate	5.94E-04	1.13E-03	1.22E-03	1.54E-03
Salivary glands	1.99E-04	2.08E-04	3.67E-04	3.87E-04
Red Marrow	9.58E-04	4.78E-04	7.84E-04	7.94E-04
Skeleton	1.12E-03	6.57E-04	1.02E-03	1.45E-03
Spleen	1.40E-02	1.01E-02	1.42E-02	1.31E-02
Testes	6.88E-03	6.09E-03	1.10E-03	1.16E-03
Thymus	4.11E-04	3.42E-04	5.31E-04	6.72E-04
Thyroid	2.91E-04	2.76E-04	4.44E-04	4.80E-04
Urinary bladder	5.57E-02	1.25E-01	1.17E-01	1.08E-01
Total body	1.54E-03	2.39E-03	2.67E-03	5.85E-03
Effective dose (mSv/MBq)	6.49E-03	1.35E-02	1.16E-02	3.13E-02

References:

1. Bratanovic, I. J.; Zhang, C.; Zhang, Z.; Kuo, H. T.; Colpo, N.; Zeisler, J.; Merkens, H.; Uribe, C.; Lin, K. S.; Bénard, F., A radiotracer for molecular imaging and therapy of gastrin-releasing peptide receptor–positive prostate cancer. *Journal of Nuclear Medicine* **2022,** *63* (3), 424-430.
